# Dietary *Lonicera japonica* supplementation modulates cecal gut microbial composition and metabolomic profiles in weaned piglets

**DOI:** 10.3389/fvets.2026.1804735

**Published:** 2026-06-09

**Authors:** Xia Zhang, Hailong Huo, Lijuan Hu, Fuhua Yang, Xiaojing Hu, Yunze Deng, Caiping Feng, Haizhen Wang, Jinlong Huo

**Affiliations:** 1 Department of Biological and Food Engineering, Lyuliang University, Lvliang, Shanxi, China; 2 Yunnan Open University, Kunming, Yunnan, China; 3 College of Animal Science and Technology, Shanxi Agricultural University, Taiyuan, Shanxi, China; 4 Yunnan Academy of Animal Husbandry and Veterinary Sciences, Kunming, Yunnan, China; 5 College of Animal Science and Technology, Yunnan Agricultural University, Kunming, Yunnan, China

**Keywords:** cecal contents, *Lonicera japonica* supplementation, metabolomics, metagenomics, weaned piglets

## Abstract

Weaning is a critical developmental stage in piglets and is often associated with intestinal dysbiosis, metabolic disturbances, and impaired gut barrier function. Phytogenic feed additives have emerged as promising natural alternatives to antibiotics for improving gut health. *Lonicera japonica*, a traditional medicinal and edible plant rich in bioactive compounds, exhibits well-documented antimicrobial, antioxidant, and immunomodulatory properties; however, its effects on the gut microbiota-metabolite axis in weaned piglets remain poorly understood. In this study, weaned piglets were fed either a basal diet (control group) or a *Lonicera japonica*-supplemented diet (experimental group). Cecal contents were collected for metagenomic sequencing to characterize gut microbial composition and for untargeted LC–MS-based metabolomic profiling. Functional pathway enrichment and microbe-metabolite correlation network analyses were conducted to elucidate potential mechanisms. *Lonicera japonica* supplementation significantly improved evenness in terms of microbial species richness and reshaped microbial community structure, characterized by the enrichment of beneficial taxa, including *Firmicutes* and *Eubacterium coprostanoligenes*, and a concomitant reduction in opportunistic pathogens such as *Proteobacteria* and *Escherichia coli*. KEGG pathway analysis revealed the upregulation of microbial pathways related to translation, replication, and energy metabolism, alongside the downregulation of stress-response-associated pathways. Metabolomic profiling demonstrated distinct metabolic signatures between groups, with elevated levels of unsaturated fatty acids, amino acid derivatives, and organic acids, and reduced bile acid intermediates in the *Lonicera japonica*-treated piglets. Correlation network analysis further revealed strong positive correlations between SCFA-producing bacteria and beneficial metabolites, underscoring a reinforced microbiota-metabolite axis. Collectively, these findings indicate that *Lonicera japonica* supplementation promotes a healthier and more stable gut ecosystem in weaned piglets through coordinated modulation of microbial composition, functional potential, and metabolic outputs. This study provides novel insights into microbiota-metabolite interactions underlying phytogenic interventions and supports the use of *Lonicera japonica* as a natural feed additive to enhance intestinal health and resilience during weaning.

## 1 Introduction

The weaning period represents one of the most critical and vulnerable stages in the life of piglets. Following weaning, piglets are exposed to multiple challenges, including the abrupt transition from sow’s milk to solid feed, environmental and social stressors, and an immature immune system. These stressors frequently result in reduced feed intake, growth retardation, diarrhea, and increased mortality rates (1). From a physiological perspective, weaning stress damages the intestinal barrier, alters gut microbiota, and disrupts lipid, carbohydrate, and amino acid metabolism, leading to energy metabolic imbalance (2). Lipid metabolism dysregulation is strongly linked to energy imbalance, intestinal dysfunction, and weakened immunity, and is a key factor in reduced growth after weaning (3). The gut microbiota plays a central role in the regulating of lipid metabolism by influencing fatty acid absorption and synthesis, modulating bile acid metabolism (4). Alterations in the relative abundance of specific bacterial taxa, such as *Firmicutes* and *Bacteroidetes*, can directly affect energy harvest efficiency and fat deposition (5, 6). In addition, short-chain fatty acids (SCFAs) produced by the gut microbiota-such as acetate, propionate, and butyrate-play an important role in regulating lipid metabolism in the liver and adipose tissue (7). Consequently, targeting the gut microbiota to alleviate lipid metabolic disturbances in weaned piglets has emerged as a major focus in animal nutrition research.

Among natural plant-derived feed additives, *Lonicera japonica* has attracted increasing scientific attention due to its multifunctional biological properties. *Lonicera japonica* is rich in bioactive compounds, including chlorogenic acid, luteolin, quercetin, flavonoids, saponins, and phenolic acids (8). These constituents exhibit potent antioxidant, anti-inflammatory, antibacterial, and immunomodulatory activities. They act mainly by inhibiting of key signaling pathways such as the nuclear factor kappa B (NF-kB) and mitogen-activated protein kinase (MAPK) pathways, while also enhancing endogenous antioxidant defense systems (9). Previous studies have shown that dietary supplementation with *Lonicera japonica* extracts significantly improves intestinal morphology, increases the relative abundance of lactic acid bacteria, and suppresses pathogenic microorganisms in pigs (10). Consistently, *Lonicera japonica* supplementation has been reported to promote beneficial microbial populations while inhibiting pathogens such as *Escherichia coli*, contributing to improved intestinal microbial homeostasis and feed conversion efficiency (11). In addition to its microbiota-modulating effects, *Lonicera japonica* has been demonstrated to upregulate the expression of intestinal epithelial tight junction proteins, including zonula occludens-1 (ZO-1), occludin, and claudin-1. This enhancement of barrier function subsequently alleviates intestinal inflammation and oxidative stress in the host (12, 13). Its major active components, chlorogenic acid and luteolin, further contribute to intestinal immune regulation by promoting secretory immunoglobulin A (slgA) production and modulating the Treg/Th17 cell balance, thus maintaining intestinal immune homeostasis (14). Moreover, *Lonicera japonica* has been implicated in regulating hepatic and plasma lipid profiles through its influence on fatty acid metabolism and cholesterol biosynthesis pathways (15). Studies in aquatic species have shown that dietary *Lonicera japonica* supplementation enhances immune function, improves lipid metabolic balance, and stabilizes intestinal microbial communities (16). Collectively, these findings suggest that *Lonicera japonica* may exert beneficial effects on lipid metabolism via modulation of the gut microbiota-host metabolic axis.

However, despite accumulating evidence supporting the functional application of *Lonicera japonica* in livestock and poultry production, its precise role and underlying mechanisms in regulating lipid metabolism in weaned piglets remain incompletely understood. In particular, it remains unclear whether *Lonicera japonica* can restore metabolic homeostasis by reshaping the intestinal microecological environment and modulating lipid metabolic pathways. Given the complexity of the interactions among gut microorganisms, metabolites, and host physiological processes, single-omics approaches are often insufficient to comprehensively elucidate these mechanisms. Therefore, an integrative multi-omics strategy is warranted (17). The combined application of metagenomics and untargeted metabolomics provides a powerful framework for deciphering the mechanistic links between microbial functions and metabolic processes. Metagenomic analysis enables comprehensive profiling of gut microbial taxonomic composition and functional gene potential. Untargeted metabolomics allows systematic characterization of host- and microbiota-derived metabolic alterations. Integrating these complementary approaches enables the identification of changes in the gut microbiota and their links to host metabolic remodeling (18). Recent studies employing integrated metagenomic-metabolomic approaches have demonstrated that weaning stress induces profound alterations in gut microbial composition and associated metabolic pathways in piglets. (19). Notably, interventions with probiotics or plant-derived bioactive compounds have been shown to partially or fully reverse these adverse effects, underscoring the pivotal role of the gut microbiota in regulating fat deposition and energy metabolic homeostasis (20).

Here, we systematically investigated the effects of *Lonicera japonica* supplementation on intestinal microbial structure, functional gene composition, and lipid metabolism in weaned piglets using an integrated metagenomic and untargeted metabolomic approach. The specific objectives were to: (1) elucidate the regulatory effects of *Lonicera japonica* supplementation on the composition and functional metabolic potential of the intestinal microbiota; (2) identify key metabolites and metabolic pathways associated with lipid metabolism; and (3) construct a microbiota-metabolite interaction network to elucidate the potential mechanisms by which *Lonicera japonica* modulates lipid metabolism. This study provides a theoretical basis for utilizing *Lonicera japonica* as a sustainable feed additive for weaned piglets. It also yields novel multi-omics insights into how natural plant compounds in host metabolic homeostasis.

## 2 Materials and methods

### 2.1 Experimental animals and feeding management

All piglets were routinely vaccinated and dewormed throughout the experimental period. The experimental and control groups were raised in different enclosures under standard commercial husbandry conditions. Piglets had ad libitum access to feed, and water were fed three times daily at 07:00, 12:00, and 17:00. All management, housing, and vaccination procedures were conducted in accordance with standard practices for commercial pig production. A total of 20 healthy 21-day-old weaned Duroc × Landrace × Yorkshire crossbred piglets with similar initial body weights (no significant difference; *p* > 0.05) were selected for the study. Piglets were randomly assigned to two experimental groups (n = 10 per group). During the 35-day experimental period, the control group (CON) was fed a basal diet, whereas the experimental group (FLJ) received the same basal diet supplemented with *Lonicera japonica* flower. The basal diet was supplemented with 1,000 mg/kg dried *Lonicera japonica* powder, which was thoroughly homogenized with the feed prior to administration. The powder contained 7.87% moisture, while the concentrations of the principal bioactive were quantified as 1.535% chlorogenic acid and 0.0541% luteolin. The ingredient composition and nutrient levels of the basal diet (air-dried basis) are presented in Supplementary Table S1.

### 2.2 Sample collection

On day 35 of the experimental period, six piglets were randomly selected from each group (provided that there was no significant difference in weight at the start of the experiment) as target animals for multi-omics analysis. The piglets were anesthetized and then humanely euthanized by exsanguination via the jugular vein. All of the research was authorized and approved by the ethical committee of Lyuliang University (SHANXI Lvliang, China). Every effort was made to minimize suffering. Immediately following euthanasia (within 2–3 min), the abdominal cavity was opened and the cecum was rapidly isolated. Subsequently, the entire dissection and content-collection procedure was performed on an ice plate under strictly controlled low-temperature conditions, thereby minimizing potential metabolic alterations and microbial perturbations. The collected cecal contents were promptly aliquoted into sterile cryovials, snap-frozen in liquid nitrogen, and stored at −80 °C until further analysis.

### 2.3 Growth performance, diarrhea rate and serum biochemical index detection

Body weight was recorded for each piglet at both the beginning and the end of the experiment, while feed intake per pen was continuously monitored throughout the trial. Subsequently, Average daily gain (ADG), average daily feed intake (ADFI), and feed-to-gain ratio (F/G) were calculated. Fecal consistency was visually assessed daily using a standardized five-point scoring system, as previously described (21), thereby enabling calculation of the diarrhea rate. In addition, piglet health status and mortality were monitored and recorded daily throughout the experiment period. Following overnight fasting, 5 mL of blood was collected from the anterior vena cava into anticoagulant tubes and analyzed using an automated hematology analyzer (Mindray BC-30 Vet, China). Additionally, 10 mL of blood was collected for serum preparation, centrifuged at 3,500 rpm for 10 min, and stored at −20 °C until subsequent analysis. Serum biochemical parameters were determined using an automated biochemical analyzer (DOTOP-8018).

### 2.4 Metagenomic sequencing and data analysis

Total microbial DNA was extracted from cecal content samples (n = 6 per group) following the manufacturer’s protocols. DNA was extracted using the CretMag™ Power Soil DNA Kit (27100-4-EP, CretBiotech, China) in accordance with the manufacturer’s instructions. For mechanical lysis, samples were homogenized using a TGrinder H24 tissue homogenizer (OSE-TH-01, Tiangen Biotech), with bead-beating parameters set at 6.0 m/s for 30 s, followed by a 30 s pause, for a total of two cycles. Subsequently, the quality and concentration of the extracted DNA were assessed, and qualified DNA samples were stored at −80 °C until further processing. DNA libraries were constructed, fragmented, and subjected to quality control before sequencing. Qualified libraries were sequenced on the NovaSeq 6,000 platform (Illumina, San Diego, CA, USA) using a 150-bp paired-end strategy. Raw sequencing reads were processed using Trimmomatic (version 0.40) (22) to remove adapter sequences and low-quality reads, and host-derived sequences were filtered using BMTagger (version 3.101), yielding high-quality clean reads for downstream analyses. The resulting high-quality, host-depleted reads were *de novo* assembled using MEGAHIT with the parameters—min-contig-len 500—k-min 21—k-max 141—k-step 12. Contigs with lengths ≥ 500 bp were retained for downstream analyses. Assembly quality was subsequently evaluated using QUAST with default parameters. Protein-coding genes were predicted from assembled contigs using Prodigal in metagenomic mode (−p meta), thereby generating both nucleotide and amino acid sequences. Thereafter, predicted gene sequences from all samples were pooled and clustered using CD-HIT with the parameters -c 0.95 -aS 0.9 -g 1 -d 0 to construct a non-redundant gene catalog. Finally, clean reads from each sample were mapped back to this catalog using Bowtie 2 in end-to-end mode with the parameter—very-sensitive, and gene abundance profiles were quantified using SAMtools and featureCounts with the parameters -p -t CDS -g ID, thereby enabling downstream functional and statistical analyses.

Taxonomic annotation was performed to determine the relative abundances of microbial taxa at the phylum, genus, and species levels. Functional annotation of the representative sequences was performed using DIAMOND v0.9.19 (23) with an E-value threshold of 1e-5. Specifically, sequence annotation was conducted against the NCBI NR database using the blastp algorithm, while taxonomic classification was carried out against the eggNOG database, and pathway-level annotation was performed using the Kyoto Encyclopedia of Genes and Genomes (KEGG) database (version 94.2). Stacked bar plots were generated using the ggplot2 package (version 3.4.4) within the tidyverse framework in R (version 4.3.1). Differences in microbial community composition between groups were assessed using non-parametric statistical methods. Alpha diversity indices were compared using the Kruskal-Wallis test, while beta diversity was evaluated based on Bray–Curtis dissimilarity, with group differences tested by permutational multivariate analysis of variance (PERMANOVA). Principal coordinate analysis (PCoA) was conducted to visualize microbial community similarities among samples. Functional profiling was conducted based on KEGG (24) pathway annotation. Pathway enrichment analysis was performed using the clusterProfiler package (version 4.10.0) in R (version 4.3.1). Differentially expressed genes were identified based on an absolute log_2_ fold change > 1 and an adjusted *p* < 0.05. Significantly enriched pathways were determined using a hypergeometric test with Benjamini-Hochberg correction, and pathways with adjusted *p* < 0.05 were considered statistically significant. Enrichment results were visualized using clusterProfiler and ggplot2. Linear discriminant analysis effect size (LEfSe) (25) was applied to identify differentially abundant taxa between groups. The Kruskal-Wallis test was used to detect taxa with significant differences followed by linear discriminant analysis (LDA) to estimate effect size. Taxa with an LDA score (log_10_) ≥ 2.0 and *p* < 0.05 were considered significantly enriched. Results were visualized as LDA score histograms.

### 2.5 Untargeted metabolomics sequencing and data analysis

Samples were separated using an Agilent 1,290 UHPLC system equipped with a HILIC column, with the column temperature maintained at 25 °C, a flow rate of 0.5 mL/min, and an injection volume of 2 μL. The mobile phase comprised solvent A (water containing 25 mM ammonium acetate and 25 mM ammonia) and solvent B (acetonitrile). Subsequently, following UHPLC separation, the analytes were subjected to mass spectrometric detection using an AB Triple TOF 6600 system (AB SCIEX), operating in both positive and negative electrospray ionization (ESI) modes. Thereby enabling comprehensive metabolic coverage. The ESI source parameters were configured as follows: Gas1 = 60 psi, Gas2 = 60 psi, CUR = 30 psi, ion source temperature = 600 °C, and spray voltage = ±5,500 V. Raw LC–MS/MS data were processed using XCMS (v3.12.0) (26, 27) for peak detection, retention time alignment, and signal normalization. Metabolite annotation was performed by matching accurate mass, retention time, and MS/MS fragmentation patterns against the Human Metabolome Database (HMDB) (28), KEGG, and METLIN databases. QC-based signal correction and missing value imputation were applied to ensure high-quality datasets for subsequent analyses.

Multivariate statistical analyses, including principal component analysis (PCA) and orthogonal partial least squares discriminant analysis (OPLS-DA), were conducted using MetaboAnalyst (29) to evaluate metabolic differences between groups. Differential metabolites were identified based on variable importance in projection (VIP) scores (VIP > 1.0), univariate statistical analysis (*p* < 0.05), and fold change thresholds. KEGG pathway enrichment analysis was subsequently performed to identify significantly altered metabolic pathways associated with treatment. Heatmaps, volcano plots, and pathway enrichment visualizations were generated using the R package ggplot2 (version 3.4.4) and pheatmap (version 1.0.12).

### 2.6 Statistical analysis

Data normality was assessed *a priori* prior to statistical analyses to ensure appropriate test selection. Intergroup comparisons were conducted using Student’s t-test for normally distributed data, whereas differential metabolites were identified using the Wilcoxon rank-sum test, with false discovery rate (FDR) correction applied to account for multiple testing. Microbe–metabolite associations were evaluated using Spearman’s rank correlation coefficient. All statistical analyses were performed using R (v4.3.2) and GraphPad Prism (v10.2.1), with statistical significance defined as a two-tailed *p*-value or FDR < 0.05.

## 3 Results

### 3.1 Growth performance, diarrhea rate, and serum biochemical results

At baseline, no significant difference in initial body weight was detected between the two groups (*p* > 0.05). By contrast, on days 14 and 35, piglets in the FLJ group exhibited significantly higher body weight than those in the CON group (*p* < 0.05), accompanied by a concomitant increase in average daily gain (ADG) (*p* < 0.05) (Supplementary Table S2). Regarding serum biochemical parameters, the FLJ group demonstrated significantly elevated total protein (TP) and globulin (GLO) levels relative to the CON group (*p* < 0.05), whereas alanine aminotransferase (ALT) and total bilirubin (TBIL) were significantly reduced (*p* < 0.05). No significant differences were detected in the remaining biochemical indices (*p* > 0.05) (Supplementary Table S3). Collectively, these findings suggested that FLJ supplementation effectively improved growth performance in piglets, while being associated with improved immune-related biochemical profiles and ameliorated hepatic function, without exerting a discernible effect on feed intake.

### 3.2 Overview of metagenomic sequencing data

After quality control and filtering, high-quality metagenomic sequencing data were obtained for all samples. Detailed sequencing statistics are summarized in Supplementary Table S4. In total, 531,773,478 raw reads were generated, corresponding to 76.00 Gb of raw data. Following quality filtering, 70.33 Gb of clean data were retained, with each sample yielding more than 5.33 Gb of valid sequences. The average GC content of the clean reads was 48.26%, and the Q20 and Q30 values reached 97.55 and 93.68%, respectively, indicating high sequencing accuracy and data reliability.

To further evaluate assembly quality for downstream analyses, the distribution of contig lengths was examined for each sample (Supplementary Figure S1). The majority of assembled contigs ranged from 500 to 1,000 bp, accounting for an average of 67.04% in the FLJ group and 65.25% in the CON group. Contigs with lengths between 1,000 and 1,500 bp represented 15.14 and 15.41% of the assemblies in the FLJ and CON groups, respectively. Overall, these results indicate that the metagenomic assemblies were of sufficient quality for subsequent microbial diversity and functional analyses.

### 3.3 Microbial diversity analysis of cecal contents

**Figure 1:**
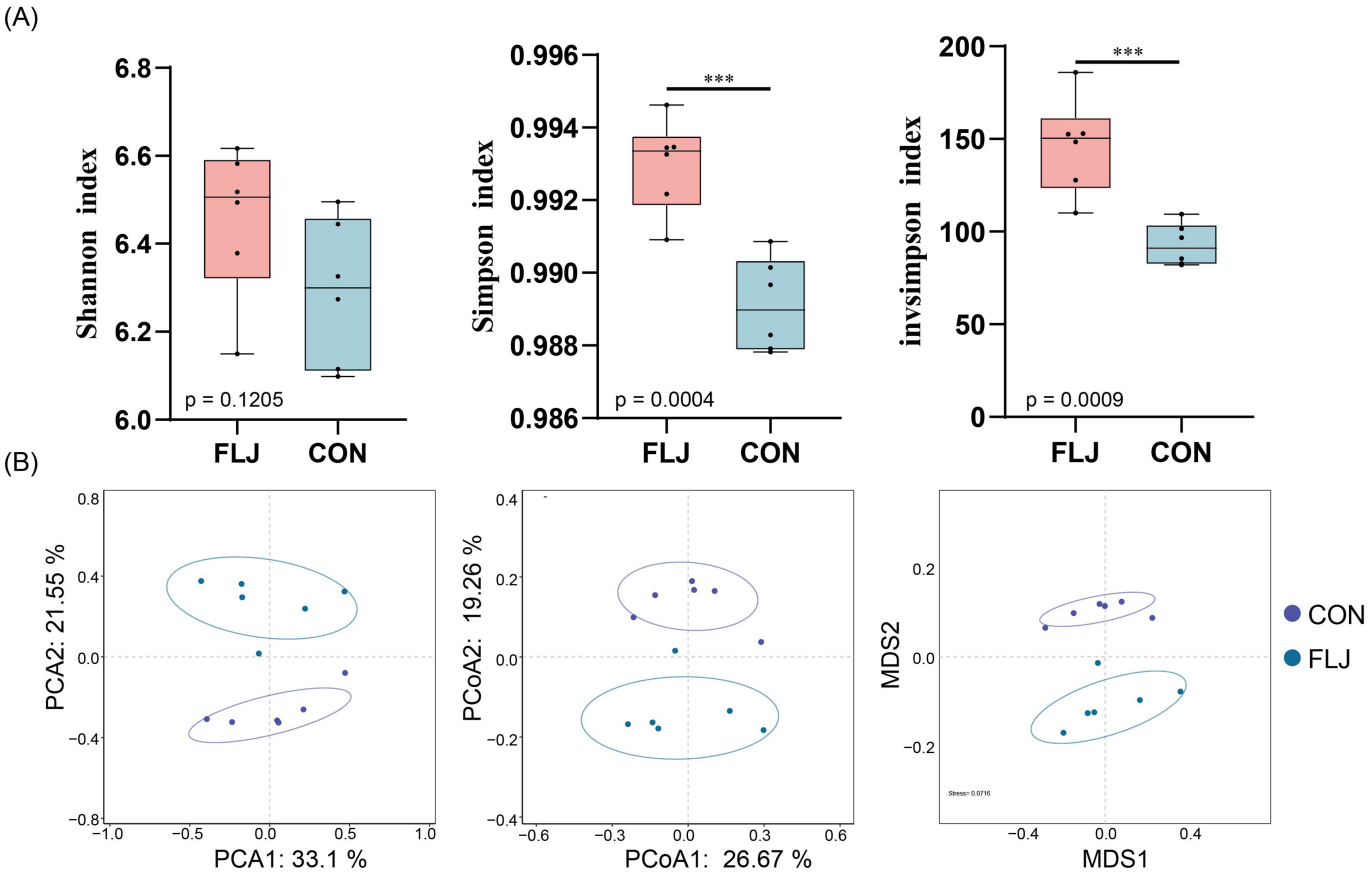
Diversity of cecal content community composition: **(A)** Alpha diversity (Shannon index, Simpson index, inverse Simpson index). **(B)** Beta diversity analyses based on principal coordinates analysis (PCoA), principal component analysis (PCA), and non-metric multidimensional scaling (NMDS).

Alpha diversity analysis was performed to evaluate microbial richness, diversity, and evenness within individual samples. The Shannon diversity index showed no significant difference in overall species richness between the FLJ and CON groups (*p* = 0.1205), although the FLJ group exhibited a numerically higher diversity trend with reduced intra-group variability. In contrast, the Simpson index revealed a significant increase in community evenness in the FLJ group compared with the CON group (*p* = 0.0004). Consistently, the Inverse Simpson index was significantly higher in the FLJ group (*p* = 0.0009), indicating an increased effective species number and reduced dominance of individual taxa. Collectively, these alpha diversity indices showed that there was no significant difference in species richness (Shannon index) among the FLJ groups; however, microbial evenness was significantly improved. (Figure 1A). Detailed alpha diversity metrics are provided in Supplementary Table S5.

Beta diversity analysis was conducted to assess differences in microbial community composition between groups. Principal coordinate analysis (PCoA) based on Bray–Curtis dissimilarity revealed a clear separation between the FLJ and CON groups, with the first two axes (PC1 and PC2) explaining 22.67 and 19.26% of the total variation, respectively. Principal component analysis (PCA) showed a similar clustering pattern, with PC1 and PC2 accounting for 33.10 and 21.55% of the variance. Non-metric multidimensional scaling (NMDS) analysis further supported the group-wise separation (Figure 1B).

Based on the Bray-Curtis distance calculated from relative abundance at the species level, PERMANOVA showed a significant difference in gut microbiota structure between the FLJ and CON groups (*F* = 2.34, R^2^ = 0.190, *p* = 0.028). The intragroup dispersion test was not significant (*F* = 0.16, *p* = 0.698), indicating that the community differences were mainly driven by differences in intergroup community composition (centroid) rather than intragroup dispersion (Supplementary Table S6). Together, these analyses indicate that the overall structure of the cecal microbiota differed between groups.

### 3.4 Bacterial community structure in cecal contents

**Figure 2:**
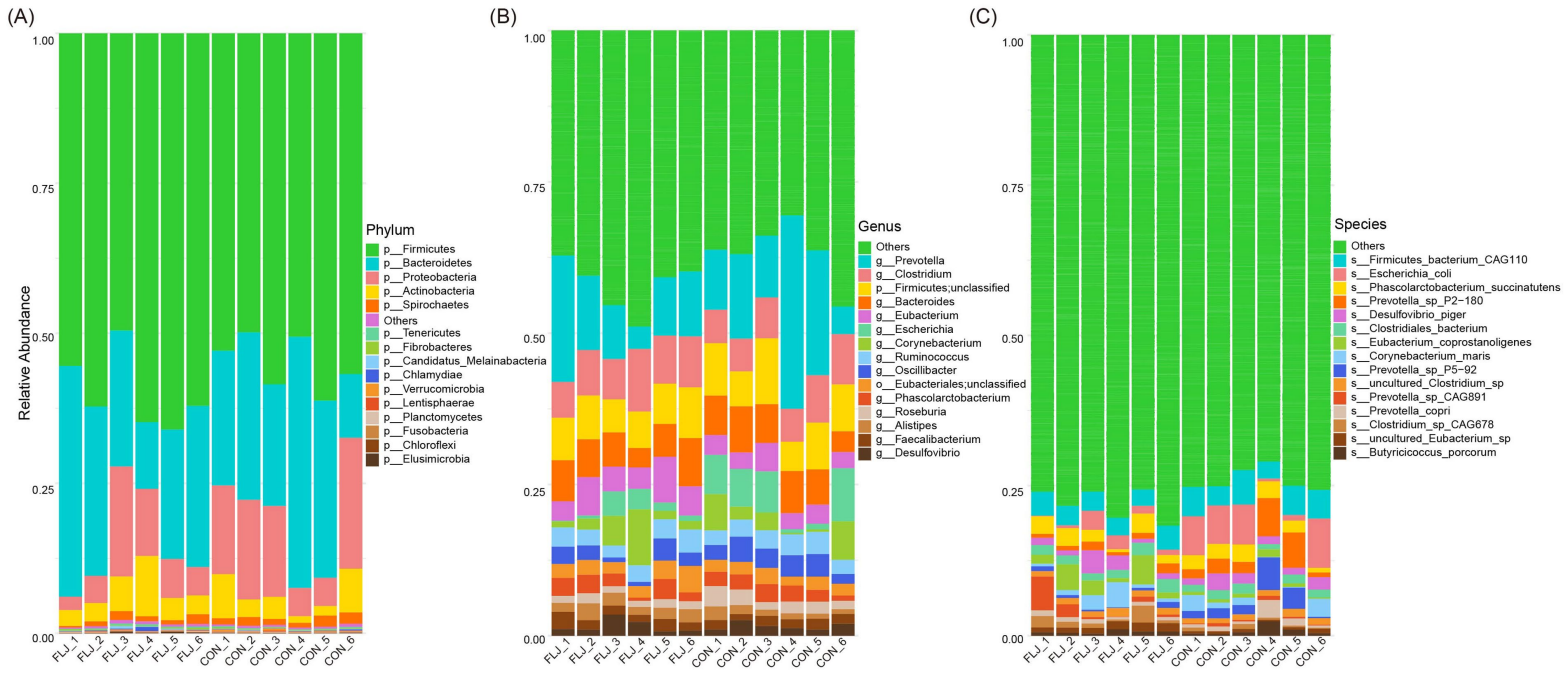
Microbial community composition at phylum, genus, and species levels. **(A–C)** Stacked bar plots showing the relative abundance of microbial communities at the phylum **(A)**, genus **(B)**, and species **(C)** levels in the FLJ group (*n* = 6) and the CON group (*n* = 6).

Taxonomic profiling of the metagenomic data identified a total of 14,385 bacterial species, classified into 147 phyla, 233 classes, 387 orders, 757 families, and 2,754 genera. To characterize differences in microbial composition between groups, relative abundances were analyzed at the phylum, genus, and species levels. At the phylum level, the cecal microbiota of both groups was dominated by *Firmicutes*, *Bacteroidota*, *Proteobacteria*, *Actinobacteria*, and *Spirochaetes* (Figure 2A). Compared with the CON group, the FLJ group exhibited higher relative abundances of *Firmicutes* (60.00% vs. 54.95%) and *Actinobacteria* (4.75% vs. 4.01%), accompanied by lower abundances of *Bacteroidota* (24.83% vs. 25.47%) and Proteobacteria (7.90% vs. 12.93%). At the genus level, the predominant taxa included *Prevotella*, *Clostridium*, *Bacteroides*, *Eubacterium*, and *Escherichia* (Figure 2B). The FLJ group showed higher relative abundances of *Clostridium* and *Eubacterium*, whereas *Prevotella*, *Bacteroides*, and *Escherichia* were reduced compared with the CON group. At the species level, dominant taxa included *Firmicutes bacterium CAG110*, *Escherichia coli*, *Phascolarctobacterium succinatutens*, *Prevotella* sp. P2-180, and *Desulfovibrio piger* (Figure 2C). The relative abundances of these dominant species were generally lower in the FLJ group than in the CON group, reflecting differences in microbial composition at the species level.

### 3.5 Analysis of differentially abundant microbes between FLJ and CON groups

**Figure 3:**
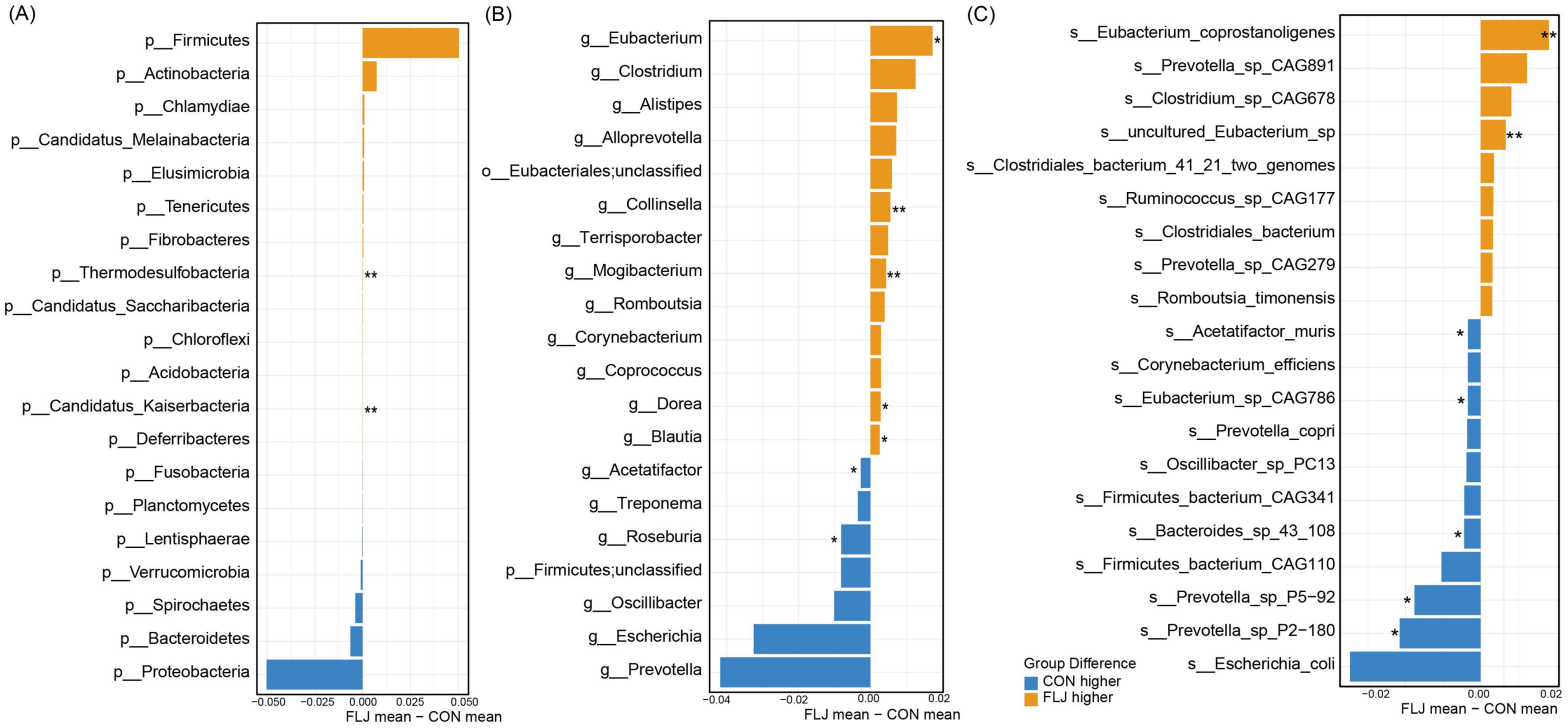
Analysis of microbial community differences among groups **(A–C)**. Differences in bacterial species between the FLJ and CON groups at the phylum level, the genus level, species level (Wilcoxon rank-sum test). ** indicates *p* < 0.01, * indicates *p* < 0.05.

At the phylum level, the FLJ group showed higher relative abundances of *Firmicutes* and *Actinobacteria*, while lower abundances of *Bacteroidota* and *Proteobacteria* compared with the CON group, and differences were also observed in several low-abundance phyla, including *Thermodesulfobacteria* and *p_Candidatus_Kaiserbacteria* (Figure 3A). At the genus level, the dominant genera in the FLJ group were *Eubacterium*, *Alloprevotella*, and *Lachnospiraceae*, whereas those in the control group (CON) were Prevotella, Escherichia, and Bacteroides. Higher relative abundances of Eubacterium and other beneficial genera while reducing potentially pathogenic genera such as Escherichia and several other genera in the FLJ group, whereas *Prevotella* and Escherichia were enriched in the CON group. Significant differences were also detected for *Collinsella*, *Mogibacterium*, *Eubacterium*, *Dorea,* and *Blautia* (Figure 3B). At the species level, *Eubacterium_coprostanoligenes* and *Firmicutes_bacterium* CAG110 were enriched in the FLJ group, whereas *Escherichia_coli* and P*revotella_sp_P2-180* were enriched in the CON group, with *Eubacterium_coprostanoligenes* and *uncultured_Eubacterium_sp* exhibiting the highest LDA scores (Figure 3C). Overall, *Lonicera japonica* treatment markedly modulated the cecal microbiota, promoting beneficial bacteria while suppressing potential pathogens, and exerted multi-level regulatory effects on non-dominant microbial taxa.

### 3.6 Functional potential of the cecal microbiota revealed by KEGG enrichment analysis

**Figure 4:**
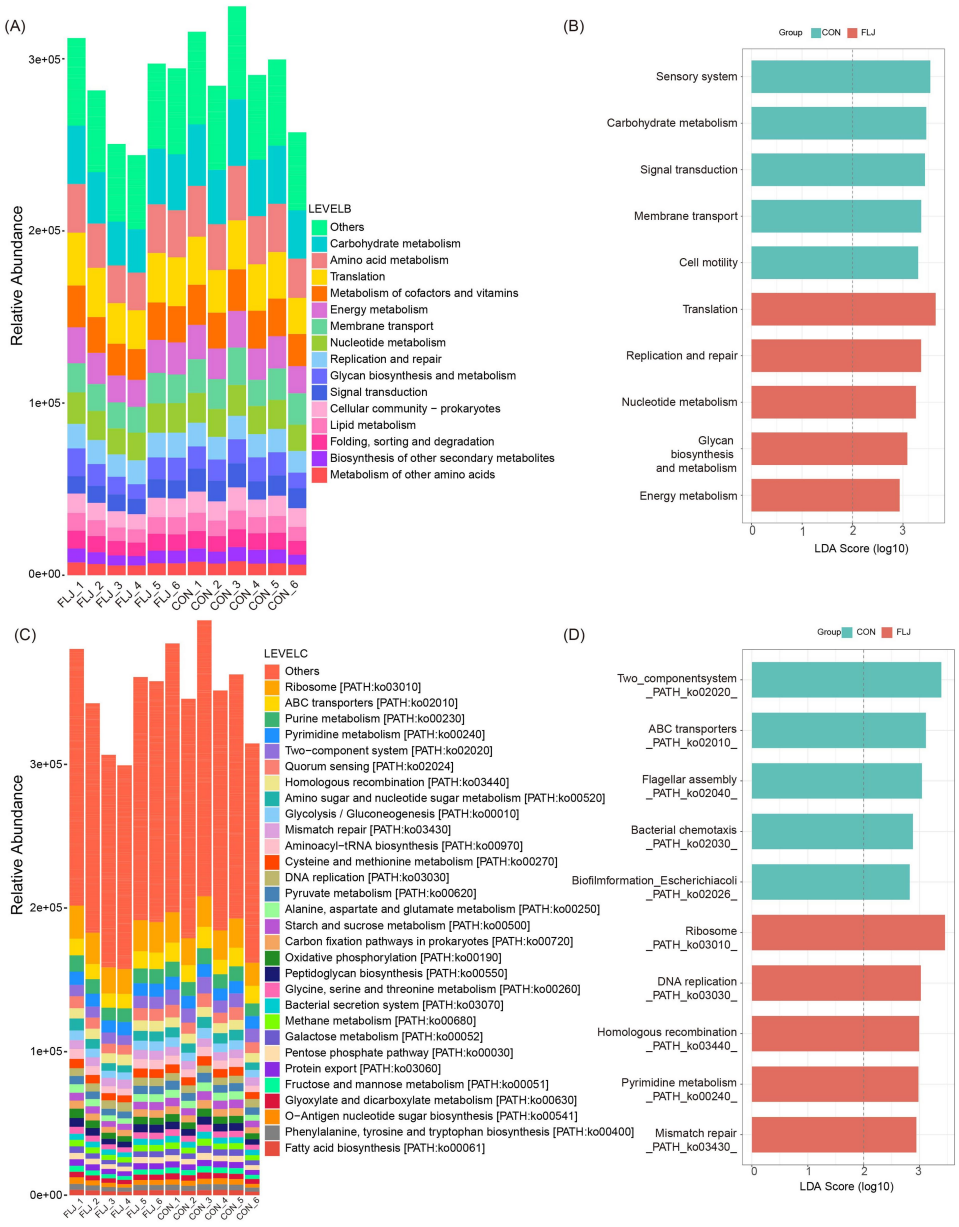
Functional pathway differences of the cecal microbiota between the FLJ and CON groups based on KEGG analysis. **(A)** Relative abundance of KEGG functional categories at LEVEL2. **(B)** Differentially enriched KEGG LEVEL2 pathways between the FLJ and CON groups identified by LEfSe analysis (LDA score >2.0, *p* < 0.05). **(C)** Relative abundance of KEGG functional pathways at LEVEL3. **(D)** Differentially enriched KEGG LEVEL3 pathways between the FLJ and CON groups identified by LEfSe analysis (LDA score > 2.0, *p* < 0.05).

To explore differences in the functional potential of the cecal microbiota between groups, metagenomic reads were annotated against the KEGG database, and functional enrichment analyses were performed. At KEGG level 2, microbial functions were primarily associated with metabolism-related categories, including carbohydrate metabolism, amino acid metabolism, energy metabolism, and nucleotide metabolism (Figure 4A). LEfSe analysis identified distinct functional signatures between the FLJ and CON groups at this level (Figure 4B). Pathways related to translation, replication and repair, nucleotide metabolism, glycan biosynthesis and metabolism, and energy metabolism were enriched in the FLJ group. In contrast, functions associated with membrane transport, signal transduction, carbohydrate metabolism, cell motility, and sensory systems were enriched in the CON group. At KEGG level 3, more refined pathway analysis further supported these functional shifts (Figure 4C). The FLJ group was enriched in pathways involved in ribosome biogenesis, DNA replication, homologous recombination, pyrimidine metabolism, and mismatch repair (Figure 4D). Conversely, the CON group showed enrichment in two-component systems, ABC transporters, flagellar assembly, bacterial chemotaxis, and biofilm formation, including pathways associated with *Escherichia coli*. Overall, KEGG functional enrichment analysis revealed distinct differences in microbial functional profiles between the FLJ and CON groups. These differences were characterized by enrichment of biosynthesis- and replication-related pathways in the FLJ group and enrichment of transport- and motility-related pathways in the CON group.

### 3.7 Composition of metabolites

After database matching and compound annotation, a total of 97 metabolites were identified across all samples. These metabolites were classified according to their chemical taxonomy, and their overall distribution is summarized in Supplementary Figure S2. Among the identified metabolites, lipids and lipid-like molecules constituted the largest category, accounting for approximately one-third of the total metabolite pool, followed by organic acids and derivatives, which together represented another substantial proportion. Other metabolite classes, including organic heterocyclic compounds, benzenes, organic oxygen compounds, and phenylpropanoids and polyketides, were present at moderate levels, while nucleotides, alkaloids, lignans, and sulfur-containing compounds accounted for relatively minor fractions. At a more detailed classification level, fatty acyls, steroids and steroid derivatives, prenol lipids, and glycerophospholipids were the predominant subclasses within the lipid category, whereas carboxylic acids and derivatives represented the major component of organic acids. Overall, the metabolite composition was characterized by a predominance of lipid-and organic acid-related compounds, which were the main metabolite classes detected in the cecal contents.

### 3.8 Principal component analysis of metabolites

**Figure 5:**
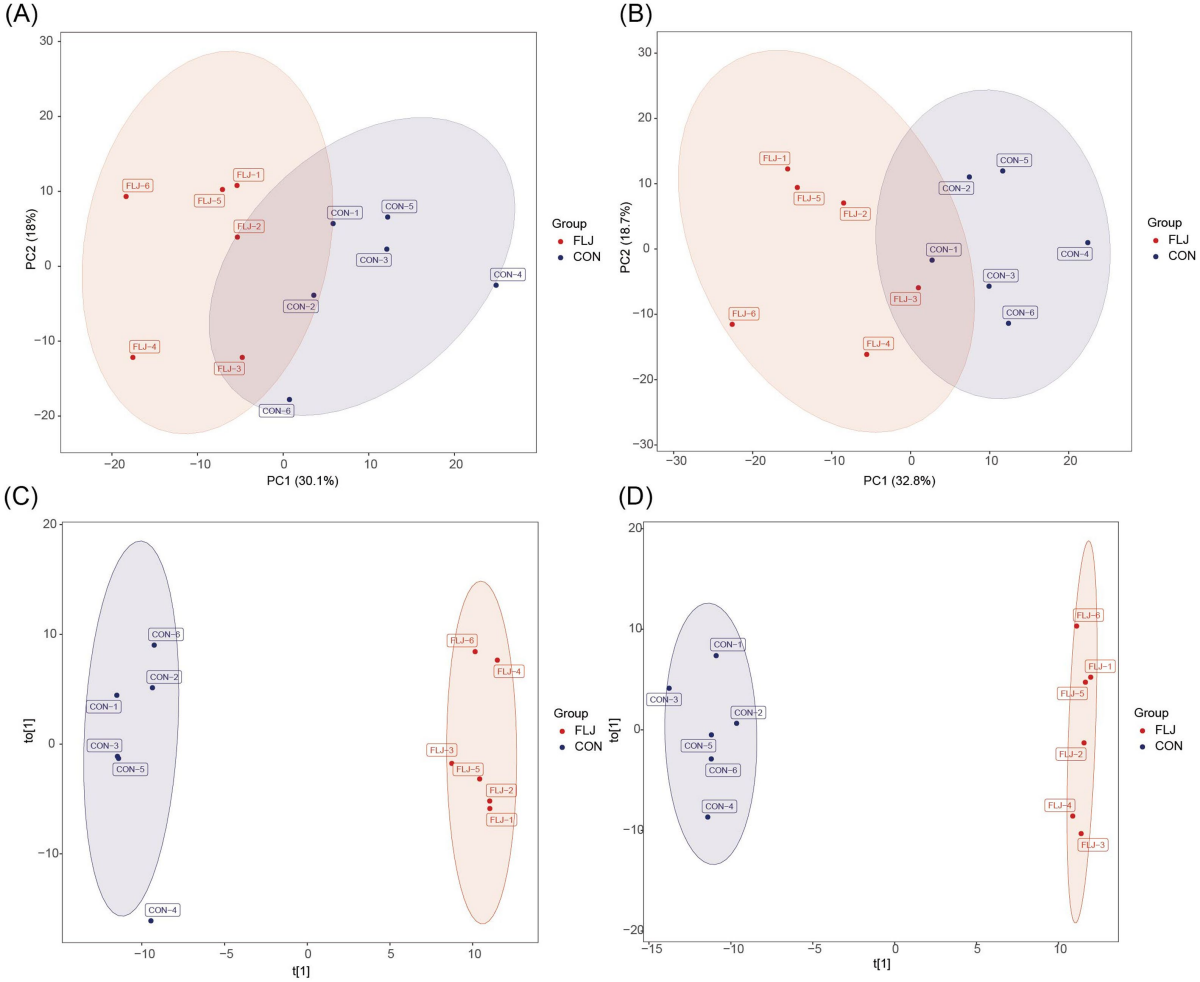
PCA and OPLS-DA of cecal metabolites in weaned piglets. **(A,B)** PCA score plots in positive and negative ion modes, respectively. **(C,D)** OPLS-DA score plots in positive and negative ion modes, respectively, showing group-level separation between the FLJ and CON groups.

Principal component analysis (PCA) was applied to assess global metabolic differences between the FLJ and CON groups. In positive ion mode, the first two principal components (PC1 and PC2) explained 30.1 and 18.0% of the total variance, respectively, whereas in negative ion mode they accounted for 32.8 and 18.7% of the variance (Figures 5A,B). PCA score plots showed a tendency toward separation between the two groups in both ionization modes, reflecting differences in overall metabolic profiles. To further evaluate group discrimination, orthogonal partial least squares discriminant analysis (OPLS-DA) was performed. The OPLS-DA models for both positive and negative ion modes were validated using 200 permutation tests. As demonstrated by the results, the Q^2^ values of all permuted models were consistently lower than those of the corresponding original models, thereby indicating that the observed discriminatory performance was unlikely to arise from random permutations. More importantly, the regression lines of the Q^2^ values intersected the Y-axis at negative intercepts (Q^2^ = −0.236 for negative ion mode and −0.1283 for positive ion mode), further substantiating model validity and effectively excluding overfitting. The OPLS-DA score plots demonstrated a clear separation between the FLJ and CON groups in both positive and negative ion modes (Figures 5C,D), indicating distinct metabolite profiles between groups. Together, the PCA and OPLS-DA analyses revealed clear group-level differences in the global metabolite profiles of cecal contents, providing a basis for subsequent identification of differential metabolites and pathway analyses.

### 3.9 Identification of differentially abundant metabolites

**Figure 6:**
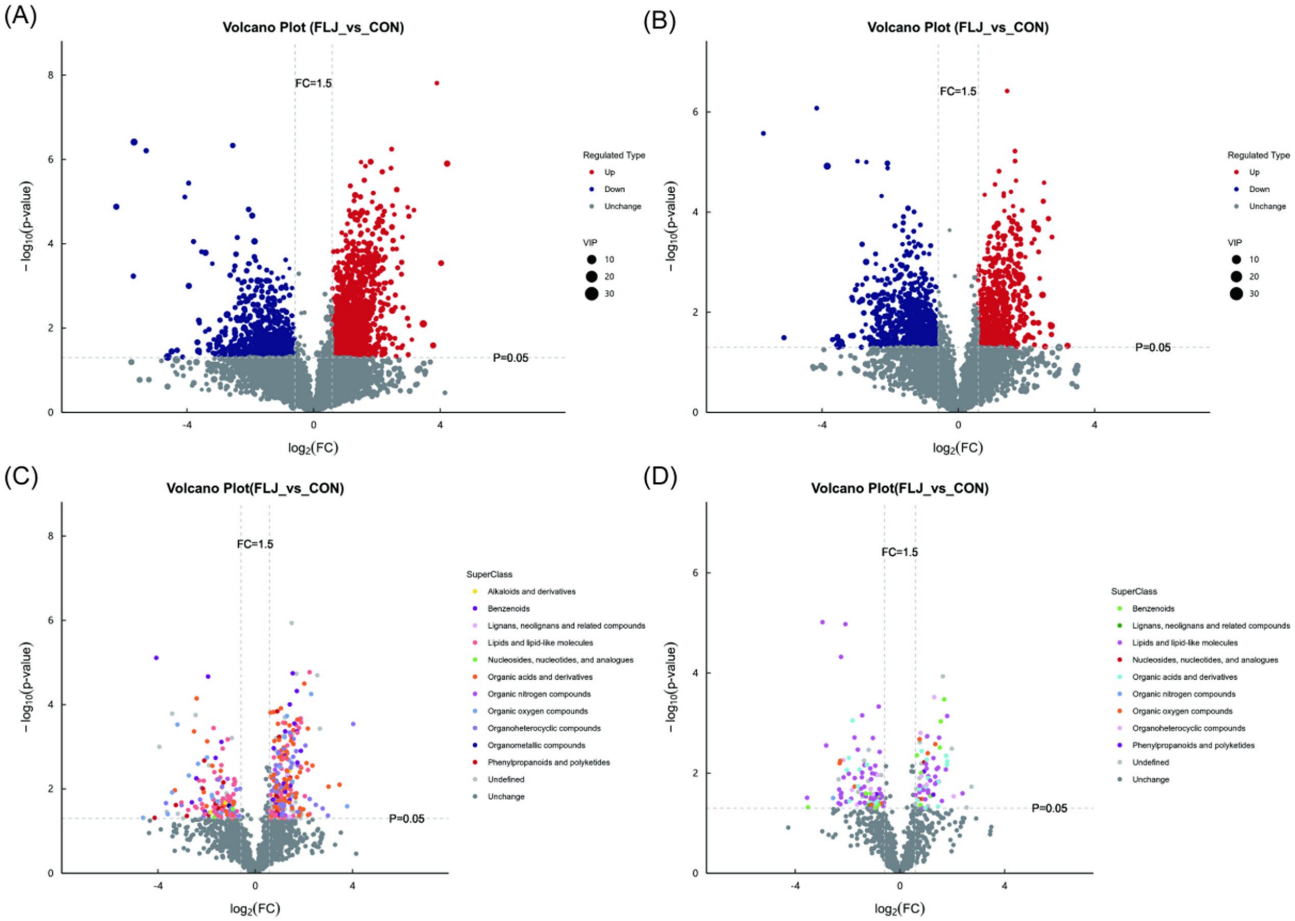
Volcano plots of differential metabolic features and annotated metabolites in the cecal contents of weaned piglets. **(A,B)** Volcano plots of differential metabolic features in positive and negative ion modes, respectively, identified based on variable importance in projection (VIP) scores. **(C,D)** Volcano plots of annotated differential metabolites in positive and negative ion modes, categorized according to chemical superclasses. Differential features and metabolites were defined by VIP > 1.0, fold change (FC) > 1.5, and *p* < 0.05.

Untargeted metabolomic analysis detected a total of 9,151 metabolic features in positive ion mode, among which 1,203 features were significantly increased, and 663 were decreased in the FLJ group compared with the CON group. In negative ion mode, 8,367 metabolic features were detected, including 672 upregulated and 706 downregulated features (Figures 6A,B). Following metabolite annotation, 16.8% of the detected features in positive ion mode were successfully identified as known metabolites. Among these annotated metabolites, 459 compounds (5.0%) were associated with lipid metabolism, and 420 compounds (4.5%) were classified as organic acids and derivatives. In negative ion mode, 9.0% of the detected features were annotated, including 303 lipid-related metabolites (3.6%) and 152 organic acids and derivatives (1.8%) (Figures 6C,D). Collectively, these results revealed extensive differences in the cecal metabolite profiles between the FLJ and CON groups, with a large proportion of differential metabolites related to lipid metabolism and organic acid pathways.

**Figure 7:**
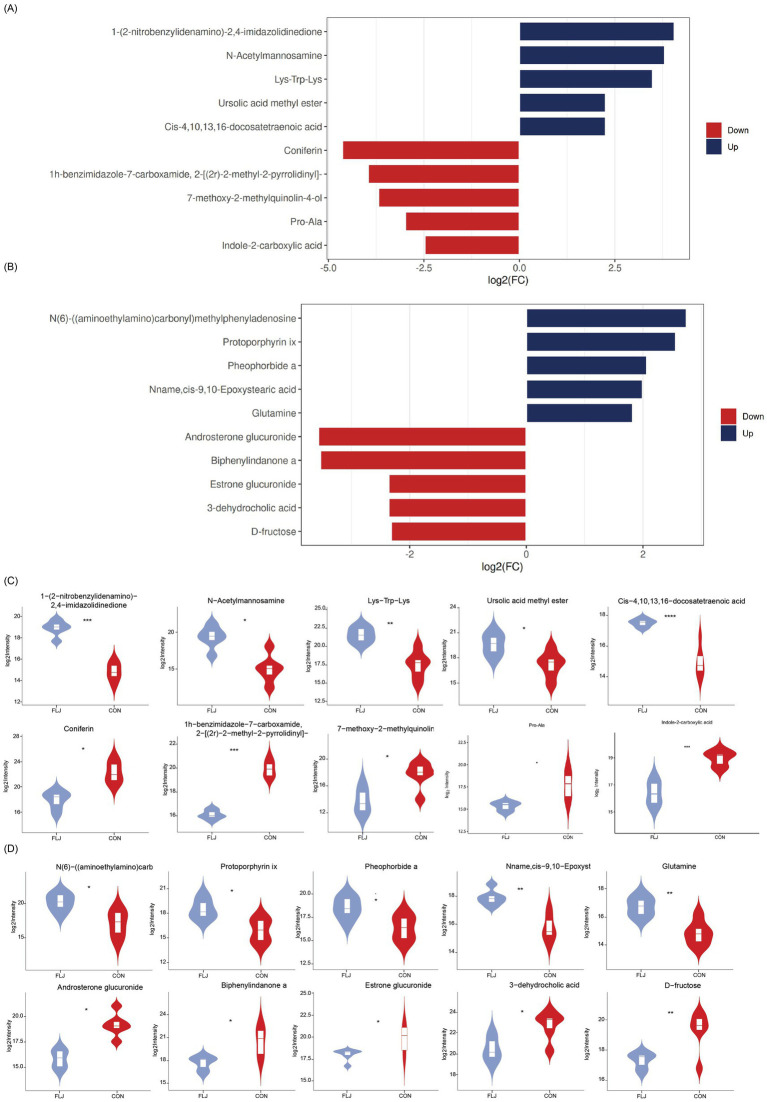
Comparison of the top five significantly upregulated and downregulated metabolites between the FLJ and CON groups. **(A,B)** Relative abundance of the top five upregulated and downregulated metabolites identified in positive and negative ion modes, respectively. **(C,D)** Statistical comparison of the relative abundance of the top five upregulated and downregulated metabolites between the FLJ and CON groups in positive and negative ion modes. Data are presented as mean ± SEM. *p < 0.05, **p < 0.01, ***p < 0.001.

Further characterization of differential metabolites revealed pronounced metabolic alterations induced by *Lonicera japonica* supplementation in both positive and negative ion modes. In positive ion mode, metabolites with higher abundances in the FLJ group were predominantly enriched in amino acid derivatives, peptides, unsaturated fatty acids, prostaglandins, and triterpenoids, whereas metabolites with lower abundance were mainly associated with sugars, amino acid derivatives, polyphenols, alkaloids, and prostaglandin-related compounds (Supplementary Table S7). In negative ion mode, metabolites enriched in the FLJ group were primarily involved in fatty acid metabolism, amino acid metabolism, steroid hormone biosynthesis, porphyrin metabolism, and eicosanoid signaling. In contrast, metabolites enriched in the CON group were largely associated with bile acid metabolism, steroid hormone conjugation, carbohydrate metabolism, and xenobiotic-related pathways (Supplementary Table S8). Comparative analysis of the top five significantly enriched and depleted metabolites in both ion modes further confirmed consistent group-level differences in metabolite abundance between the FLJ and CON groups (Figures 7A,B). Violin plot analysis revealed clear distributional separation of representative differential metabolites between groups, demonstrating the robustness of the metabolomic data (Figures 7C,D). Overall, differential metabolite analysis showed coordinated changes in multiple metabolic categories between groups.

### 3.10 Cluster analysis of differential metabolites

**Figure 8:**
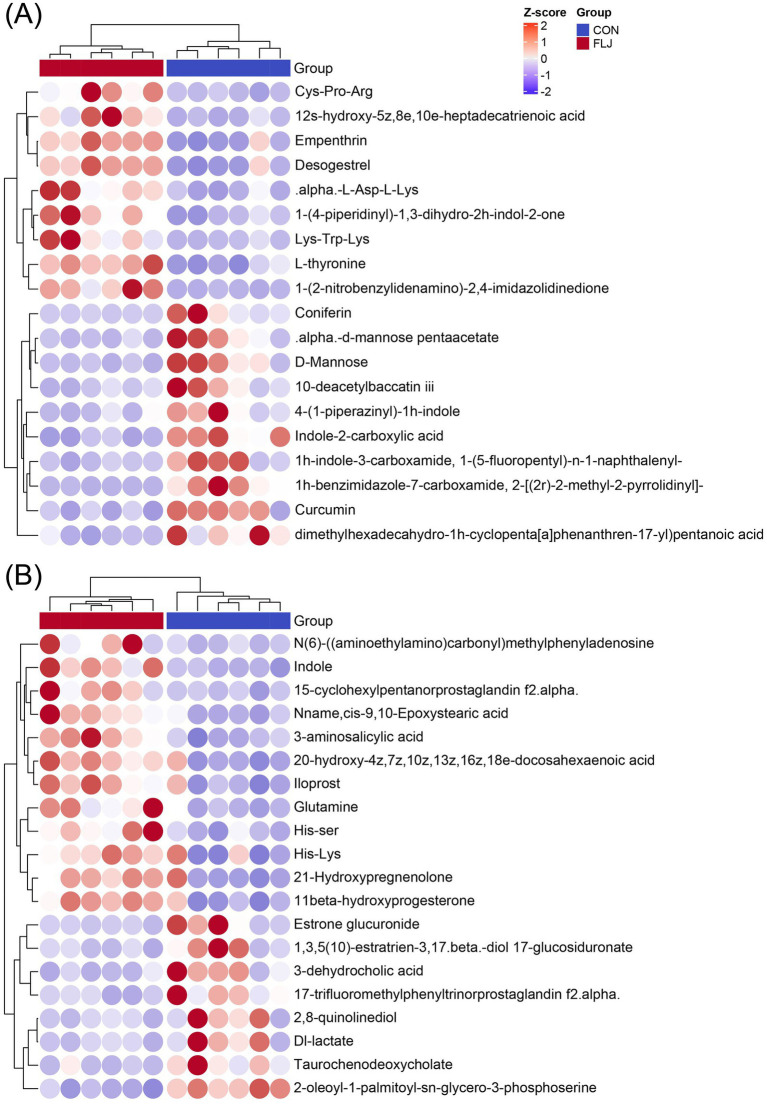
Cluster heat map of differential metabolites between the FLJ and CON groups. **(A)** Hierarchical clustering heat map of the top 10 differential metabolites in the positive ion mode. **(B)** Hierarchical clustering heat map of the top 10 differential metabolites in the negative ion mode. Differential metabolites were selected based on VIP > 1.0 and *p* < 0.05. Color intensity represents normalized relative abundance (*Z*-score) of metabolites.

Based on positive ion mode data, a hierarchical clustering heatmap was constructed using the top 10 differential metabolites ranked by VIP value. These metabolites were classified into six major chemical categories (Figure 8A). Notably, several lipid-related and heterocyclic compounds, including arachidonic acid-derived metabolites, steroid hormone precursors, and L-thyronine, exhibited distinct abundance patterns between the FLJ and CON groups. In addition, three bioactive short peptides (e.g., Cys-Pro-Arg and Lys-Trp-Lys) were identified, showing clear group-specific clustering patterns, indicating pronounced differences in peptide-related metabolism. Similarly, hierarchical clustering analysis based on negative ion mode data revealed clear separation between the FLJ and CON groups using the top 10 differential metabolites (Figure 8B). These metabolites mainly belonged to steroid hormones, prostaglandins, phospholipids, amino acids and their derivatives, quinoline compounds, and microcystins, with a predominance of acidic and highly polar metabolites. Among them, multiple prostaglandin derivatives (e.g., 15-cyclohexylpentanorprostaglandin F2α and 17-trifluoromethylphenyltrinorprostaglandin F2α), epoxy fatty acids (cis-9,10-epoxystearic acid), and phosphatidylserine-related lipids (2-oleoyl-1-palmitoyl-sn-glycero-3-phosphoserine) displayed marked differences in relative abundance between groups. Overall, hierarchical clustering analysis demonstrated that differential metabolites in both ionization modes exhibited consistent and distinct grouping patterns between the FLJ and CON groups, with lipid- and inflammation-associated metabolites emerging as prominent clustered features.

### 3.11 KEGG pathway enrichment analysis of differential metabolites

**Figure 9:**
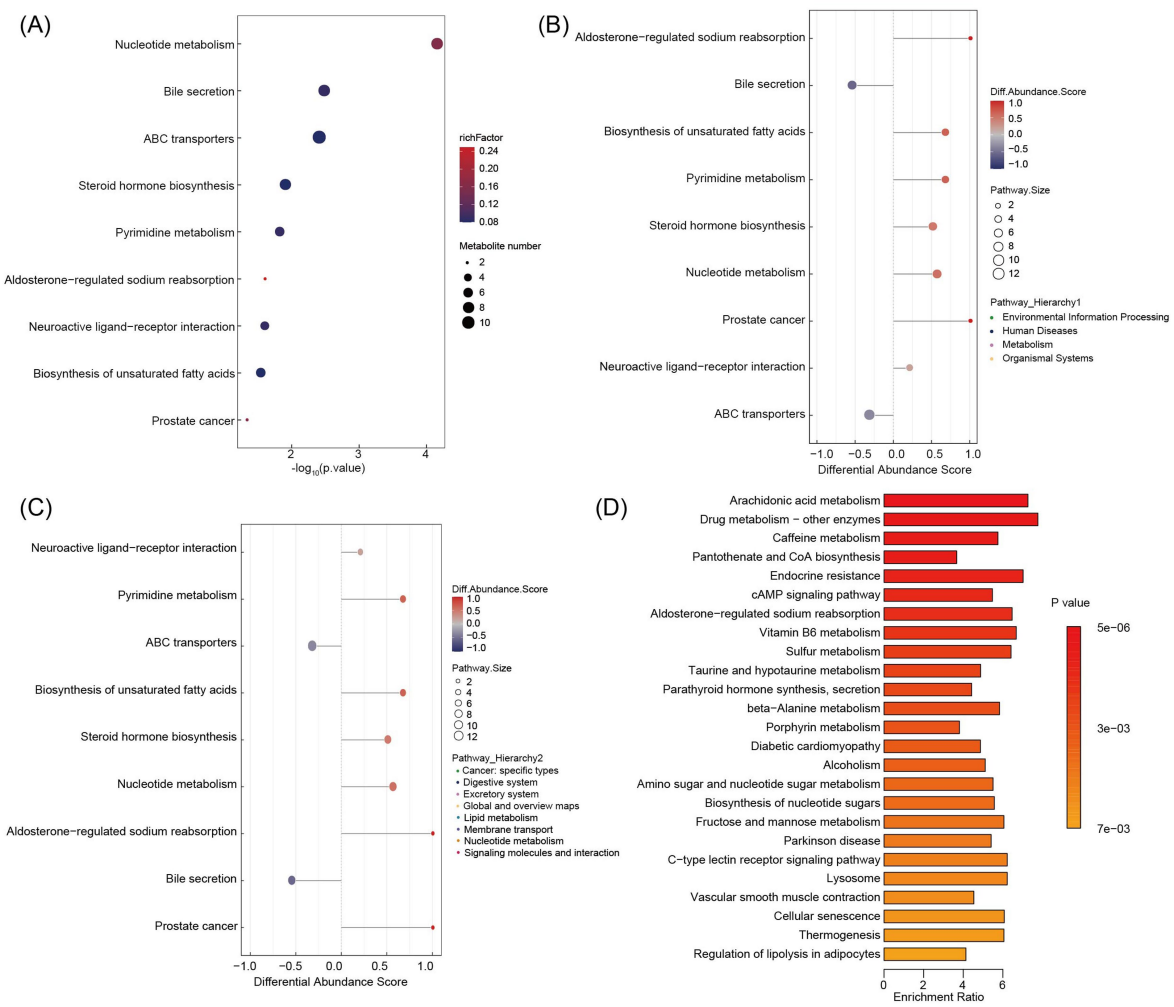
Integrated pathway enrichment and functional analysis of differential metabolites. **(A)** KEGG pathway enrichment bubble plot based on significantly differential metabolites, where bubble size represents the number of metabolites and color indicates enrichment significance. **(B,C)** DAScore plots at the primary (H1) and secondary (H2) KEGG classification levels, illustrating overall upregulation or downregulation trends of metabolic pathways between the FLJ and CON groups. **(D)** MSEA showing significantly enriched metabolic pathways associated with *Lonicera japonica* supplementation.

To comprehensively characterize the metabolic pathways affected by *Lonicera japonica* supplementation, KEGG pathway enrichment analysis was conducted based on the identified differential metabolites. Pathway enrichment was evaluated using the Rich Factor, defined as the proportion of significantly altered metabolites relative to the total number of metabolites annotated within each pathway. As shown in the bubble plot (Figure 9A), differential metabolites were predominantly enriched in pathways associated with lipid metabolism (biosynthesis of unsaturated fatty acids, steroid hormone biosynthesis, bile secretion), as well as pathways related to nucleotide and energy metabolism (nucleotide metabolism and pyrimidine metabolism) and membrane transport processes (ABC transporters).

KEGG annotation identified the top nine significantly enriched pathways, involving 39 differential metabolites. These pathways could be broadly categorized into three functional groups: lipid metabolism-related pathways, nucleotide metabolism-related pathways, and transport or signaling-associated pathways. Among them, the biosynthesis of unsaturated fatty acids was enriched with six metabolites, five of which were upregulated, including cis-4,7,10,13,16,19-docosahexaenoic acid, cis-7,10,13,16,19-docosapentaenoic acid, arachidic acid, behenic acid, and erucic acid, while linoleic acid was downregulated. Steroid hormone biosynthesis pathway was enriched with eight metabolites, showing increased levels of hydrocortisone, aldosterone, progesterone, 21-hydroxypregnenolone, and 11β-hydroxyprogesterone, along with decreased levels of androsterone glucuronide and estrone glucuronide. In addition, the bile secretion pathway was characterized primarily by downregulated levels of bile acid-related metabolites, including chenodeoxycholate, deoxycholic acid, taurolithocholic acid sulfate, and taurochenodeoxycholate. Pathways related to nucleotide metabolism and transport were also significantly affected. Pyrimidine metabolism and nucleotide metabolism showed enrichment of multiple upregulated nucleosides and nucleobases, including uridine, thymine, uracil, and adenosine, whereas cytidine and xanthine were downregulated. The ABC transporter pathways involved both upregulated metabolites (uridine, glutamine, adenosine, and His-Lys) and downregulated carbohydrate-related substrates, such as D-mannose, D-fructose, maltotriose, and N-acetylglucosamine, indicating alterations in substrate transport and utilization.

To further visualize pathway-level changes, a Differential Abundance Score (DAScore) analysis was performed. At the primary KEGG classification level, pathways related to metabolism-particularly lipid metabolism, nucleotide metabolism, and energy metabolism-showed an overall upregulation trend in the FLJ group compared with the CON group, whereas pathways associated with neuroactive signaling exhibited a relative downregulation (Figure 9B). At the secondary classification level, biosynthesis of unsaturated fatty acids and steroid hormone biosynthesis were consistently grouped under lipid metabolism, further supporting the metabolite-level enrichment results (Figure 9C).

In addition, metabolic set enrichment analysis (MSEA) revealed that the most significantly enriched pathways in the FLJ group were primarily associated with lipid metabolic regulation. These included cofactor-providing pathways such as pantothenate and coenzyme A biosynthesis, signaling pathways involved in lipid metabolism regulation, including the cAMP signaling pathway and aldosterone-regulated sodium reabsorption, as well as pathways associated with lipid-related metabolic disorders, such as endocrine resistance and diabetic cardiomyopathy (Figure 9D). Collectively, these results indicate that *Lonicera japonica* supplementation is associated with multi-level modulation of lipid-related metabolic pathways, accompanied by coordinated changes in nucleotide metabolism and membrane transport processes in the cecal metabolic profile of weaned piglets.

### 3.12 Correlation network between gut microbiota and metabolites

**Figure 10:**
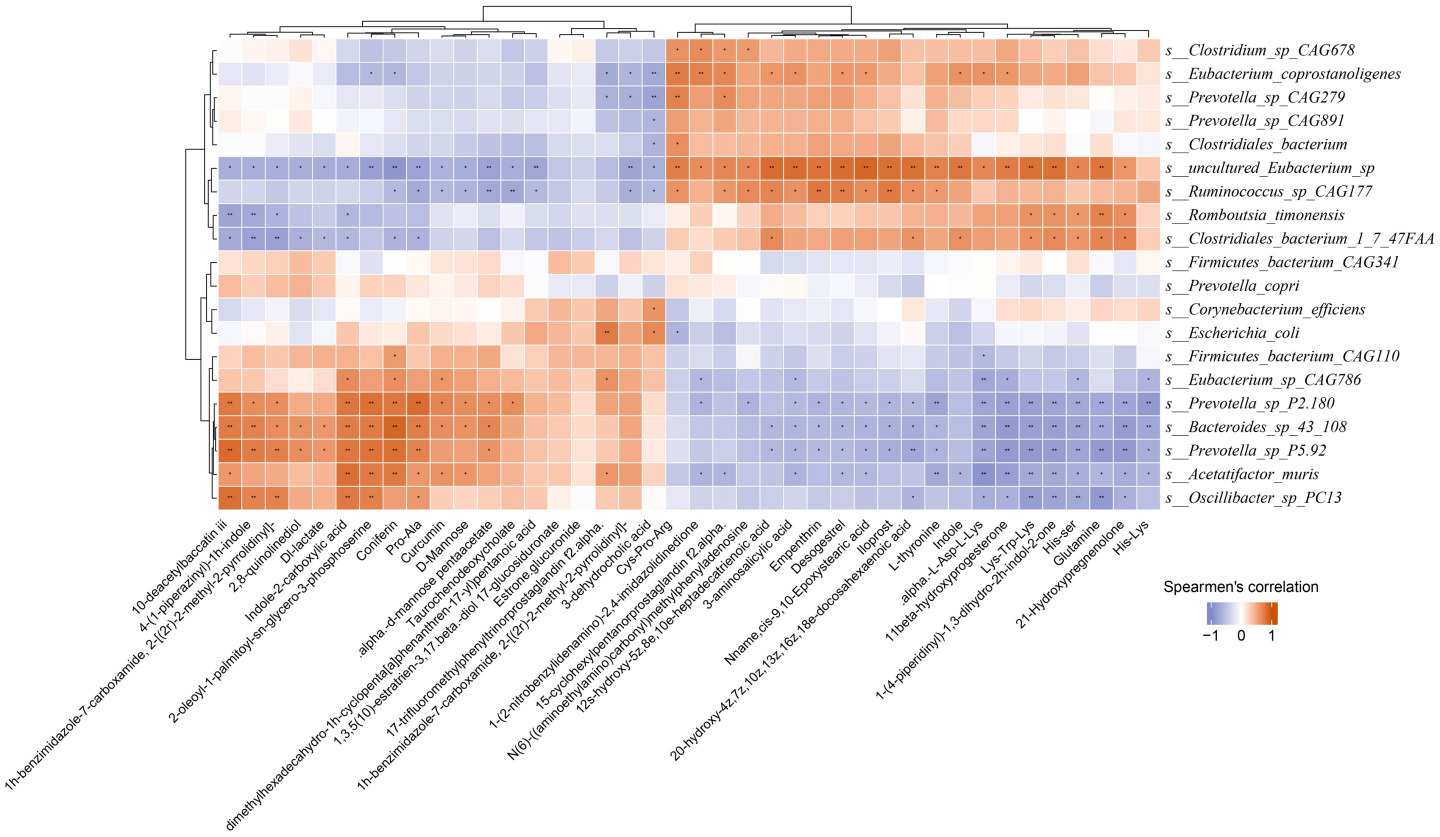
Correlation network between gut microbiota and metabolites. Spearman correlation heatmap between the top 20 differential bacterial species and metabolites.

To further explore the associations between gut microbial taxa and metabolic alterations following *Lonicera japonica* supplementation, Spearman’s correlation analysis was performed between differentially abundant microbial species and metabolites. The resulting correlation matrix was visualized as a clustered correlation heatmap (Figure 10). Overall, distinct clustering patterns were observed between bacterial taxa and metabolites, with correlation coefficients ranging from approximately −1 to 1, indicating both positive and negative associations within the gut microbiota–metabolite network. *Prevotella* sp. *CAG279*, *Prevotella* sp. *CAG891*, *Clostridium* sp. *CAG678* and *Eubacterium coprostanoligenes* exhibited strong positive correlations with multiple differential metabolites, which were mainly involved in amino acid metabolism and SCFA-related pathways. Additionally, certain members of the phylum *Firmicutes* showed negative associations with these metabolites, further highlighting group-specific differences in microbe-metabolite interaction patterns. Collectively, these results demonstrate that *Lonicera japonica* supplementation was associated with a distinct restructuring of the gut microbiota-metabolite correlation network, potentially contributing to the observed metabolic remodeling in weaned piglets.

## 4 Discussion

The present study provides comprehensive, integrated multi-omics evidence that dietary supplementation with *Lonicera japonica* profoundly reshaped the gut microbial composition and reprogrammed the cecal metabolic landscape of weaned piglets. Weaning is recognized as a critical and stressful developmental transition that disrupts gut homeostasis, often leading to microbial dysbiosis and inflammation (30, 31). In this study, *Lonicera japonica* supplementation was found to significantly increased microbial evenness, enriched beneficial bacterial taxa, enhanced microbial functional potential, and potentially induce coordinated alterations in lipid- and amino acid-related metabolic pathways. Furthermore, correlation network analysis revealed a relatively more structured and tightly coordinated microbe-metabolite interaction pattern in the treatment group, characterized by potentially strengthened associations between beneficial microbes and key metabolic pathways. Collectively, these findings support the hypothesis that *Lonicera japonica* may acts as a phytogenic functional feed additive capable of stabilizing the gut ecosystem during early-life nutritional transitions through modulation of the gut microbiota-metabolite axis (32, 33).

### 4.1 Effects of *Lonicera japonica* on gut microbial community structure

Gut microbial diversity is a key determinant of intestinal resilience and host health (34). In this study, *Lonicera japonica* supplementation was observed to significantly improved alpha diversity, particularly microbial evenness, indicating a more balanced microbial community structure. Higher microbial diversity has been consistently associated with relatively greater ecological stability and resistance to pathogenic invasion, particularly during the weaning period (35). Taxonomic profiling revealed a pronounced restructuring of the bacterial community. Specifically, the relative abundances of *Firmicutes* and *Actinobacteria* were elevated, whereas *Proteobacteria* were markedly reduced in the *Lonicera japonica* group. Within the Firmicutes phylum, the enrichment of SCFA-producing genera (such as *Faecalibacterium* and *Eubacterium*) is widely regarded as a hallmark of a more balanced and functionally robust gut ecosystem (36).

The observed increase in *Firmicutes* abundance was primarily driven by the enrichment of SCFA-producing genera within this phylum, such as *Faecalibacterium* and *Eubacterium*. These genera are believed to play important roles in maintaining mucosal integrity, providing energy for colonocytes, and modulating host immune responses (37, 38). In contrast, increased abundance of *Proteobacteria*-particularly *Escherichia coli*-is commonly considered to be a potential hallmark of gut dysbiosis and intestinal inflammation in weaned piglets (39). The observed reduction in these taxa suggests that *Lonicera japonica* may help suppress opportunistic pathogens, thereby potentially alleviating dysbiosis associated with weaning stress, potentially through direct antimicrobial activity or by enhancing colonization resistance via beneficial microbes expansion.

Notably, the enrichment of *Prevotella_sp_CAG279* and *Prevotella_sp_CAG891* suggests a potentially enhanced capacity for dietary fiber and complex carbohydrate fermentation. *Prevotella* species are key degraders of dietary fibers and phytochemicals and are frequently associated with SCFA production, improved mucosal health, and metabolic flexibility (40). Together, these microbial shifts indicate that *Lonicera japonica* supplementation may promote a gut microbial ecosystem characterized by enhanced functional redundancy and resilience, thereby supporting intestinal homeostasis during weaning stress.

### 4.2 Functional shifts in microbial metabolism

Beyond taxonomic restructuring, *Lonicera japonica* supplementation also appeared to induce substantial shifts in the functional metabolic potential of the gut microbiome. Functional annotation based on KEGG pathways revealed significant enrichment of pathways related to ribosome biogenesis, DNA replication, translation, energy metabolism, and amino acid biosynthesis in the treatment group.

These functions are typically associated with enhanced microbial growth capacity, efficient substrate utilization, and increased biosynthetic activity, which collectively reflect a more stable ecological state (41). In contrast, pathways related to bacterial chemotaxis, two-component regulatory systems, and environmental sensing and stress responses were relatively more abundant in the control group, suggesting a microbial community potentially adapted to fluctuating or stressful conditions (42).

Similar functional transitions have been reported in previous studies demonstrating that dietary phytogenic additives promote a metabolically active yet less inflammatory gut microbial environment (43). The bioactive constituents of *Lonicera japonica*, including chlorogenic acid, flavonoids, and iridoids, may directly modulate microbial metabolic activity or indirectly reshape microbial niches. These compounds are known to possess prebiotic, antioxidant, and antimicrobial properties, which may selectively stimulate beneficial taxa while constraining opportunistic bacteria. Collectively, these functional enhancements likely contribute to the reprogramming of microbial metabolic strategies and stabilization of the gut ecosystem during the weaning transition (44).

### 4.3 Remodeling of cecal metabolomic profiles

Untargeted metabolomics revealed distinct metabolic separation between the treatment and control groups, indicating substantial potential remodeling of metabolites related to lipid metabolism, organic acids, and nitrogen-containing compounds. One of the most notable findings was the significant reduction of bile acid-related metabolites, including taurochenodeoxycholate (TCDCA) and deoxycholic acid (DCA), in the *Lonicera japonica* group. As a highly hydrophobic secondary bile acid, excessive accumulation of DCA has been reported to exert cytotoxic and pro-inflammatory effects, thereby contributing to intestinal mucosal barrier disruption (45). In parallel, TCDCA, serving as a ligand for receptors such as FXR and TGR5, has been implicated in immune dysregulation when aberrantly elevated (46). Notably, the production of both metabolites is contingent upon microbial enzymatic activity. Accordingly, the concomitant reduction in DCA and TCDCA suggests that *Lonicera japonica* may not directly perturb hepatic primary bile acid synthesis, but it may act by reshaping the gut microbiota community, thereby suppressing the excessive generation of cytotoxic secondary bile acids at their microbial origin. This regulatory effect may reduce intestinal inflammation and improve overall lipid metabolism homeostasis while maintaining basic lipid absorption processes (47).

Conversely, *Lonicera japonica* supplementation was associated with an increased the abundance of unsaturated fatty acids and steroid hormone-related metabolites, which are known to potentially support epithelial repair, immune modulation, and energy homeostasis (48). Elevated levels of amino acid-derived metabolites and microbial fermentation-associated organic acids further suggest a potentially enhanced microbial fermentation capacity, consistent with the observed enrichment of *Prevotella* and *Clostridium*. Such metabolic remodeling may likely contributes to improved nutrient utilization and optimized energy metabolism, thereby potentially mitigating post-weaning stress and intestinal metabolic homeostasis during early-life development (49).

### 4.4 Microbiota-metabolite interaction network

Correlation network analysis provided deeper insight into the functional coupling between microbial taxa and metabolic outputs. Beneficial genera such as *Clostridium*, and *Eubacterium* exhibited strong positive correlations with SCFA- and amino acid-related metabolites, whereas *Escherichia* and *Corynebacterium* showed negative associations with lipid- and bile acid-related metabolites (50). These patterns align with the concept of the gut microbiota-metabolite axis, wherein dietary interventions reshape microbial communities and, consequently, their metabolic products.

SCFAs play pivotal roles in maintaining epithelial barrier integrity, regulating mucosal immunity, and modulating host energy metabolism (51). By promoting SCFA-producing taxa, *Lonicera japonica* supplementation likely enhances these protective mechanisms. Simultaneously, the attenuation of associations involving opportunistic pathogens and potentially harmful bile acid metabolites suggests that *Lonicera japonica* may interrupt deleterious microbe–metabolite feedback loops that exacerbate dysbiosis during weaning (52). This network-level reorganization reflects a more resilient, coordinated, and metabolically balanced intestinal ecosystem.

### 4.5 Implications for pig health and production

The observed microbial and metabolic alterations carry important practical implications for pig health and production performance. Post-weaning diarrhea, impaired growth, and increased morbidity remain major challenges in modern pig production systems (53). Traditional interventions often rely on antibiotics or pharmacological doses of zinc oxide, both of which face increasing regulatory restrictions and environmental concerns. In this context, phytogenic feed additives such as *Lonicera japonica* represent a promising, sustainable, and antibiotic-free alternative.

These microbial and metabolic shifts, including the regulation of SCFA and bile acid profiles, may play a role in maintaining intestinal homeostasis. Consequently, *Lonicera japonica* supplementation holds promise as a strategy to favorably influence gut health, thereby supporting better feed efficiency and growth performance (54). These findings are consistent with growing evidence that plant-derived bioactive compounds can modulate the gut microbiota-metabolite axis and improve production traits, offering an environmentally friendly and sustainable nutritional strategy for animal health management in intensive pig production systems (55).

## 5 Conclusion

This study suggests that dietary supplementation with *Lonicera japonica* can modulate the cecal gut ecosystem of weaned piglets. Integrated metagenomic and metabolomic analyses revealed a relatively increased microbial diversity, enrichment of beneficial taxa, and enhanced microbial functions related to energy, lipid, and amino acid metabolism. These microbial changes were accompanied by a relatively marked remodeling of the cecal metabolomic profile, particularly involving unsaturated fatty acids, steroid hormone–related metabolites, and bile acid-associated pathways. Correlation network analysis further showed that *Lonicera japonica* may have strengthened beneficial microbiota-metabolite interactions while potentially weakening associations linked to opportunistic pathogens, suggesting improved intestinal ecological stability. Collectively, these findings highlight the gut microbiota-metabolite axis as a potentially key mechanism underlying the beneficial effects of *Lonicera japonica* and support its potential use as a natural phytogenic feed additive to promote gut health during the post-weaning period.

## Data Availability

The datasets presented in this study can be found in online repositories. The names of the repository/repositories and accession number(s) can be found at: https://www.ncbi.nlm.nih.gov/, PRJNA1417956.

## References

[1] Campbell JM Crenshaw JD Polo J. The biological stress of early weaned piglets. J Anim Sci Biotechnol. (2013) 4:19. doi: 10.1186/2049-1891-4-19, 23631414 PMC3651348

[2] Gresse R Chaucheyras-Durand F Fleury MA Van de Wiele T Forano E Blanquet-Diot S. Gut microbiota dysbiosis in postweaning piglets: understanding the keys to health. Trends Microbiol. (2017) 25:851–73. doi: 10.1016/j.tim.2017.05.004, 28602521

[3] Li Y Guo Y Wen Z Jiang X Ma X Han X. Weaning stress perturbs gut microbiome and its metabolic profile in piglets. Sci Rep. (2018) 8:18068. doi: 10.1038/s41598-018-33649-8, 30584255 PMC6305375

[4] Xiong S. Gut-microbiota-driven lipid metabolism: mechanisms and applications in swine production. Meta. (2025) 15:248. doi: 10.3390/metabo15040248, 40278377 PMC12029090

[5] Turnbaugh PJ Ley RE Mahowald MA Magrini V Mardis ER Gordon JI. An obesity-associated gut microbiome with increased capacity for energy harvest. Nature. (2006) 444:1027–31. doi: 10.1038/nature05414, 17183312

[6] Canfora EE Meex RC Venema K Blaak EE. Gut microbial metabolites in obesity, NAFLD and T2DM. Nat Rev Endocrinol. (2019) 15:261–73. doi: 10.1038/s41574-019-0156-z, 30670819

[7] Dalile B Van Oudenhove L Vervliet B Verbeke K. The role of short-chain fatty acids in microbiota–gut–brain communication. Nat Rev Gastroenterol Hepatol. (2019) 16:461–78. doi: 10.1038/s41575-019-0157-3, 31123355

[8] Shang X Pan H Li M Miao X Ding H. *Lonicera japonica* Thunb.: ethnopharmacology, phytochemistry and pharmacology of an important traditional Chinese medicine. J Ethnopharmacol. (2011) 138:1–21. doi: 10.1016/j.jep.2011.08.016, 21864666 PMC7127058

[9] Wang L Huo B Huang L Che L Feng B Lin Y . Dietary supplementation with a mixture of herbal extracts during late gestation and lactation improves performance of sows and nursing piglets through regulation of maternal metabolism and transmission of antibodies. Front Vet Sci. (2022) 9:1026088. doi: 10.3389/fvets.2022.1026088, 36213410 PMC9538178

[10] Liu WC Pi SH Kim IH. Effects of Scutellaria baicalensis and *Lonicera japonica* extract mixture supplementation on growth performance, nutrient digestibility, blood profiles and meat quality in finishing pigs. Ital J Anim Sci. (2016) 15:446–52. doi: 10.1080/1828051X.2016.1202736, 37339054

[11] Lin WY Lin CJ Kuo TF Liang YC Chang CT Yang WC. Effect of phytogenic feed additives on gut microbiota and health in laying hen. J Chin Soc Anim Sci. (2019) 48:266.

[12] Chelakkot C Ghim J Ryu SH. Mechanisms regulating intestinal barrier integrity and its pathological implications. Exp Mol Med. (2018) 50:1–9. doi: 10.1038/s12276-018-0126-x, 30115904 PMC6095905

[13] Vancamelbeke M Vermeire S. The intestinal barrier: a fundamental role in health and disease. Expert Rev Gastroenterol Hepatol. (2017) 11:821–34. doi: 10.1080/17474124.2017.1343143, 28650209 PMC6104804

[14] Xu J Zhao X Yang S Tang M Zhao R Hu S. Chlorogenic acid and intestinal health: mechanistic insights and therapeutic applications. Food Funct. (2025) 16:4257–77. doi: 10.1039/D5FO00853K, 40357998

[15] Meng XL Zhu ZX Lu RH Li S Hu WP Qin CB . Regulation of growth performance and lipid metabolism in juvenile grass carp (*Ctenopharyngodon idella*) with honeysuckle (*Lonicera japonica*) extract. Fish Physiol Biochem. (2019) 45:1563–73. doi: 10.1007/s10695-019-00644-3, 31102099

[16] Meng XL Cao H Li H Li KK Yang GK Zhang YM . Effect of dietary honeysuckle (*Lonicera caerulea* L.) supplementation on lipid metabolism, immunity and intestinal microbiota in grass carp (*Ctenopharyngodon idellus*). Aquac Rep. (2022) 23:101063. doi: 10.1016/j.aqrep.2022.101063

[17] Hasin Y Seldin M Lusis A. Multi-omics approaches to disease. Genome Biol. (2017) 18:83. doi: 10.1186/s13059-017-1215-1, 28476144 PMC5418815

[18] Santiago-Rodriguez TM Hollister EB. Multi ‘omic data integration: a review of concepts, considerations, and approaches. Seminars Perinatol. (2021) 6:151456. doi: 10.1016/j.semperi.2021.151456, 34256961

[19] Wang W Hu H Zijlstra RT Zheng J Gänzle MG. Metagenomic reconstructions of gut microbial metabolism in weanling pigs. Microbiome. (2019) 7:48. doi: 10.1186/s40168-019-0662-1, 30914068 PMC6436221

[20] Luo C Xia B Zhong R Shen D Li J Chen L . Early-life nutrition interventions improved growth performance and intestinal health via the gut microbiota in piglets. Front Nutr. (2022) 8:783688. doi: 10.3389/fnut.2021.783688, 35047544 PMC8762325

[21] Hu C Song J You Z Luan Z Li W. Zinc oxide–montmorillonite hybrid influences diarrhea, intestinal mucosal integrity, and digestive enzyme activity in weaned pigs. Biol Trace Elem Res. (2012) 149:190–6. doi: 10.1007/s12011-012-9422-9, 22539019

[22] Bolger AM Lohse M Usadel B. Trimmomatic: a flexible trimmer for Illumina sequence data. Bioinformatics. (2014) 30:2114–20. doi: 10.1093/bioinformatics/btu170, 24695404 PMC4103590

[23] Buchfink B Xie C Huson DH. Fast and sensitive protein alignment using DIAMOND. Nat Methods. (2015) 12:59–60. doi: 10.1038/nmeth.3176, 25402007

[24] Kanehisa M Furumichi M Tanabe M Sato Y Morishima K. KEGG: new perspectives on genomes, pathways, diseases and drugs. Nucleic Acids Res. (2017) 45:D353–61. doi: 10.1093/nar/gkw1092, 27899662 PMC5210567

[25] Segata N Izard J Waldron L Gevers D Miropolsky L Garrett WS . Metagenomic biomarker discovery and explanation. Genome Biol. (2011) 12:R60. doi: 10.1186/gb-2011-12-6-r60, 21702898 PMC3218848

[26] Smith CA Want EJ O'Maille G Abagyan R Siuzdak G. XCMS: processing mass spectrometry data for metabolite profiling using nonlinear peak alignment, matching, and identification. Anal Chem. (2006) 78:779–87. doi: 10.1021/ac051437y, 16448051

[27] Wang R Yin Y Li J Wang H Lv W Gao Y . Global stable-isotope tracing metabolomics reveals system-wide metabolic alternations in aging *Drosophila*. Nat Commun. (2022) 13:3518. doi: 10.1038/s41467-022-31268-6, 35725845 PMC9209425

[28] Wishart DS Feunang YD Marcu A Guo AC Liang K Vázquez-Fresno R . HMDB 4.0: the human metabolome database for 2018. Nucleic Acids Res. (2018) 46:D608–17. doi: 10.1093/nar/gkx1089, 29140435 PMC5753273

[29] Chong J Wishart DS Xia J. Using MetaboAnalyst 4.0 for comprehensive and integrative metabolomics data analysis. Curr Protoc Bioinformatics. (2019) 68:e86. doi: 10.1002/cpbi.86, 31756036

[30] Saladrigas-García M Durán M D’Angelo M Coma J Pérez JF Martín-Orúe SM. An insight into the commercial piglet’s microbial gut colonization: from birth towards weaning. Anim Microb. (2022) 4:68. doi: 10.1186/s42523-022-00221-9, 36572944 PMC9791761

[31] Pluske JR Turpin DL Kim JC. Gastrointestinal tract (gut) health in the young pig. Anim Nutr. (2018) 4:187–96. doi: 10.1016/j.aninu.2017.12.004, 30140758 PMC6104527

[32] Liu Z Yan J Li N Zheng Z Zhang C Liu Z . Influence of *Lonicera japonica* and *Radix Puerariae* crude extracts on the growth performance, antioxidant capacity, and immunological functions of finishing pigs. Livest Sci. (2023) 270:105192. doi: 10.1016/j.livsci.2023.105192

[33] Mahmud MR Jian C Uddin MK Huhtinen M Salonen A Peltoniemi O . Impact of intestinal microbiota on growth performance of suckling and weaned piglets. Microbiol Spectrum. (2023) 11:e03744–22. doi: 10.1128/spectrum.03744-22, 37022154 PMC10269657

[34] Wijtten PJ van der Meulen J Verstegen MW. Intestinal barrier function and absorption in pigs after weaning: a review. Br J Nutr. (2011) 105:967–81. doi: 10.1017/S0007114510005660, 21303573

[35] Vasquez R Oh JK Song JH Kang DK. Gut microbiome-produced metabolites in pigs: a review on their biological functions and the influence of probiotics. J Anim Sci Technol. (2022) 64:671–95. doi: 10.5187/jast.2022.e58, 35969697 PMC9353353

[36] Papadomichelakis G Palamidi I Paraskeuas VV Giamouri E Mountzouris KC. Evaluation of a natural phytogenic formulation as an alternative to pharmaceutical zinc oxide in the diet of weaned piglets. Animals. (2023) 13:431. doi: 10.3390/ani13030431, 36766320 PMC9913353

[37] Jia H Xie Y Yi L Cheng W Song G Shi W . Comparative analysis of short-chain fatty acids and the immune barrier in cecum of Dahe pigs and Dahe black pigs. Animals. (2025) 15:920. doi: 10.3390/ani15070920, 40218314 PMC11987949

[38] Koh A De Vadder F Kovatcheva-Datchary P Bäckhed F. From dietary fiber to host physiology: short-chain fatty acids as key bacterial metabolites. Cell. (2016) 165:1332–45. doi: 10.1016/j.cell.2016.05.041, 27259147

[39] Shin NR Whon TW Bae JW. Proteobacteria: microbial signature of dysbiosis in gut microbiota. Trends Biotechnol. (2015) 33:496–503. doi: 10.1016/j.tibtech.2015.06.011, 26210164

[40] Yu SJ Morris A Kayal A Milošević I Van TT Bajagai YS . Pioneering gut health improvements in piglets with phytogenic feed additives. Appl Microbiol Biotechnol. (2024) 108:142. doi: 10.1007/s00253-023-12925-2, 38231265 PMC10794284

[41] Krautkramer KA Kreznar JH Romano KA Vivas EI Barrett-Wilt GA Rabaglia ME . Diet-microbiota interactions mediate global epigenetic programming in multiple host tissues. Mol Cell. (2016) 64:982–92. doi: 10.1016/j.molcel.2016.10.025, 27889451 PMC5227652

[42] Shi HN Walker A. Bacterial colonization and the development of intestinal defences. Can J Gastroenterol Hepatol. (2004) 18:493–500. doi: 10.1155/2004/690421, 15372112

[43] Camargo ND de Lima Cony BS da Silva MK Franceschi CH Andretta I. A systematic review and meta-analysis of the effect of phytogenic feed additives on pig performance. Livest Sci. (2023) 270:105190. doi: 10.1016/j.livsci.2023.105190

[44] Xie F Zhou M Li X Li S Ren M Wang C. Macrogenomic and Metabolomic analyses reveal mechanisms of gut microbiota and microbial metabolites in diarrhea of weaned piglets. Animals. (2024) 14:2327. doi: 10.3390/ani14162327, 39199861 PMC11350701

[45] Parathan P Mielke LA. Hostile bile limits anti-cancer immunity. Immunity. (2024) 57:834–6. doi: 10.1016/j.immuni.2024.03.006, 38599174

[46] Li L Liu C Mao W Tumen B Li P. Taurochenodeoxycholic acid inhibited AP-1 activation via stimulating glucocorticoid receptor. Molecules. (2019) 24:4513. doi: 10.3390/molecules24244513, 31835494 PMC6943563

[47] Duboc H Rajca S Rainteau D Benarous D Maubert MA Quervain E . Connecting dysbiosis, bile-acid dysmetabolism and gut inflammation in inflammatory bowel diseases. Gut. (2013) 62:531–9. doi: 10.1136/gutjnl-2012-302578, 22993202

[48] Nicholson JK Holmes E Kinross J Burcelin R Gibson G Jia W . Host-gut microbiota metabolic interactions. Science. (2012) 336:1262–7. doi: 10.1126/science.1223813, 22674330

[49] Liu Z Solano-Aguilar G Lakshman S Urban JF Zhang M Chen P . Metabolic pathway and network analysis integration for discovering the biomarkers in pig feces after a controlled fruit-vegetable dietary intervention. Food Chem. (2024) 461:140836. doi: 10.1016/j.foodchem.2024.140836, 39154458

[50] Zheng X Xu L Tang Q Shi K Wang Z Shi L . Integrated metagenomic and metabolomics profiling reveals key gut microbiota and metabolites associated with weaning stress in piglets. Genes. (2024) 15:970. doi: 10.3390/genes15080970, 39202331 PMC11354067

[51] Szabó C Kachungwa Lugata J Ortega AD. Gut health and influencing factors in pigs. Animals. (2023) 13:1350. doi: 10.3390/ani13081350, 37106913 PMC10135089

[52] Cao X Wang X Ren Y Sun Y Yang Z Ge J . *Lonicera caerulea* L. polyphenols improve short-chain fatty acid levels by reshaping the microbial structure of fermented feces in vitro. Front Microbiol. (2023) 14:1228700. doi: 10.3389/fmicb.2023.1228700, 37965545 PMC10641692

[53] Canibe N Højberg O Kongsted H Vodolazska D Lauridsen C Nielsen TS . Review on preventive measures to reduce post-weaning diarrhoea in piglets. Animals. (2022) 12:2585. doi: 10.3390/ani12192585, 36230326 PMC9558551

[54] Tang X Liu X Zhong J Fang R. Potential application of *Lonicera japonica* extracts in animal production: from the perspective of intestinal health. Front Microbiol. (2021) 12:719877. doi: 10.3389/fmicb.2021.719877, 34434181 PMC8381474

[55] Zhou T Jin Z Jiang R Li W. Gut microbiota modulation by traditional Chinese medicine: a translational strategy for metabolic dysfunction-associated steatotic liver disease. Front Pharmacol. (2025) 16:1600439. doi: 10.3389/fphar.2025.1600439, 40556760 PMC12185430

