# Supplementary material for: Dietary Lonicera japonica supplementation modulates cecal gut microbial composition and metabolomic profiles in weaned piglets
Source: Front Vet Sci. 2026 Jun 9;13:1804735. doi: 10.3389/fvets.2026.1804735 (PMC13289711; doi:10.3389/fvets.2026.1804735)
Supplement: Supplementary file 1 [file Data_Sheet_1.DOCX]

**Table**

**Supplementary Table S1 Composition and nutrient levels of the experimental diet (DM basis)**

| Ingredients | Proportion | Nutrient Levels² | Content (%) |
| --- | --- | --- | --- |
| Corn | 47.69 | DE (MJ/kg) | 14.60 |
| Fermented soybean meal | 14.00 | Crude protein | 18.50 |
| Puffed soybeans | 10.00 | calcium | 0.80 |
| Broken rice | 10.00 | Total phosphorus | 0.65 |
| Low protein Whey | 5.00 | Available phosphorus | 0.42 |
| Fish meal | 4.00 | Lysine | 1.36 |
| White sugar | 2.00 | Methionine | 0.45 |
| Bean oil | 2.50 | Threonine | 0.70 |
| *L*-lysine HCL hydrochloride | 0.42 | Tryptophan | 0.20 |
| *DL*-Methionine | 0.14 | Methionine +Cysteine | 0.75 |
| Tryptophan | 0.05 |  |  |
| Threonine | 0.20 |  |  |
| Calcium | 0.70 |  |  |
| NaCL | 0.30 |  |  |
| Ca(H_2_PO_4_)_2_ | 1.00 |  |  |
| Premix^1^ | 2.00 |  |  |
| Total | 100 |  |  |

1. The premix provided the following per kilogram of the diets: vitamin A 2200 IU, vitamin D3 220 IU, vitamin E 16 IU, vitamin K3 0.5 mg, vitamin B1 1 mg, vitamin B2 3.5 mg, vitamin B6 7 mg, vitamin B12 17.5 µg, biotin 0.05 mg, folic acid 10 mg, pantothenic acid 10 mg, choline 0.50 g, Cu 6 mg, Fe 100 mg, Zn 100 mg, Mn 4 mg, K 0.30 mg, I 0.14 mg, and Se 0.30 mg. (2) DE was a calculated value, while the others were measured values.

**Supplementary Table S2 Growth performance indicators of the FLJ and CON groups**

| Group | Initial weight/kg | 14 days weight/kg | 35 days weight/kg | ADG/kg | ADFI/kg | F/G | diarrhea rate% |
| --- | --- | --- | --- | --- | --- | --- | --- |
| FLJ | 16.64±0.41 | 25.12±0.68^a^ | 40.97±0.29^a^ | 0.69±0.01^a^ | 1.37±0.10 | 1.97±0.15 | 65.71±2.54^a^ |
| CON | 16.17±0.57 | 24.01±0.54^b^ | 38.74±0.74^b^ | 0.64±0.01^b^ | 1.38±0.08 | 2.13±0.12 | 42.86±1.68^b^ |

Note: lowercase letters indicate significant differences (*p* < 0.05).

**Supplementary Table S3 Blood biochemical parameters of the FLJ and CON groups**

| Index | FLJ | CON |
| --- | --- | --- |
| ALB(g/L) | 32.77±0.83 | 33.37 ± 1.25 |
| TP(g/L) | 62.65 ± 2.45^a^ | 57.63 ± 1.53^b^ |
| GLO(g/L) | 34.05 ± 2.15^a^ | 27.10 ± 4.15^b^ |
| A/G | 0.97 ± 0.23 | 1.27 ± 0.31 |
| Ca(mmol/L) | 2.60 ± 0.18 | 2.53 ± 0.28 |
| GLU(mmol/L) | 5.16 ± 0.79 | 6.06 ± 1.65 |
| BUN(mmol/L) | 4.14 ± 0.28 | 4.27 ± 1.46 |
| P(mmol/L) | 3.49 ± 0.16 | 3.77 ± 0.38 |
| AMY(U/L) | 1422.33 ± 118.35 | 1310.50 ± 72.84 |
| CHOL(mmol/L) | 3.01 ± 0.20 | 2.99 ± 0.25 |
| ALT(U/L) | 48.33 ± 5.16^b^ | 57.67 ± 5.16^a^ |
| TBIL(mmol/L) | 2.69 ± 0.36^b^ | 3.84 ± 0.99^a^ |
| ALP(U/L) | 252.17 ± 21.39 | 252.17 ± 21.39 |
| CRE(mmol/L) | 102.50 ± 17.66 | 108.17 ± 13.21 |
| BUN/CRE | 10.17 ± 1.60 | 9.50 ± 2.26 |
| CK(U/L) | 1104.33 ± 97.62 | 1108.67 ± 98.19 |

Note: lowercase letters indicate significant differences (*p* < 0.05).

**Supplementary Table S4 Metagenomic sequencing data**

| **Samples** | **Total_Reads** | **Raw Bases(GB)** | **Clean Bases(GB)** | **GC_Content** | **Q20** | **Q30** |
| --- | --- | --- | --- | --- | --- | --- |
| FLJ_1 | 43,919.624 | 6.2828 | 5.8068 | 48.92% | 98.30% | 94.70% |
| FLJ_2 | 49,108,624 | 7.0250 | 6.4441 | 50.90% | 98.45% | 95.28% |
| FLJ_3 | 43,798,440 | 6.2654 | 5.7711 | 49.85% | 98.36% | 94.95% |
| FLJ_4 | 45,208,418 | 6.4671 | 6.0483 | 47.43% | 98.54% | 95.45% |
| FLJ_5 | 45,911,172 | 6.5676 | 6.0056 | 47.91% | 98.42% | 95.08% |
| FLJ_6 | 40,550,278 | 5.8008 | 5.3376 | 48.98% | 98.36% | 94.93% |
| CON_1 | 45,095,116 | 6.4509 | 5.9620 | 50.40% | 96.67% | 92.24% |
| CON_2 | 41,418,000 | 5.9249 | 5.4706 | 47.51% | 96.47% | 91.85% |
| CON_3 | 40,917,622 | 5.8533 | 5.3843 | 46.43% | 96.22% | 91.31% |
| CON_4 | 43,502,166 | 6.2230 | 5.7360 | 44.61% | 96.72% | 92.31% |
| CON_5 | 46,282,478 | 6.6208 | 6.1975 | 48.42% | 97.09% | 93.10% |
| CON_6 | 46,061,540 | 6.5892 | 6.1648 | 47.70% | 97.02% | 92.93% |

**Supplementary Table S5 Alpha diversity indexes**

| **Samples** | **Shannon** | **Simpson** | **invsimpson** |
| --- | --- | --- | --- |
| FLJ_1 | 6.15 | 0.99 | 110.07 |
| FLJ_2 | 6.49 | 0.99 | 152.993 |
| FLJ_3 | 6.62 | 0.99 | 148.40 |
| FLJ_4 | 6.52 | 0.99 | 152.57 |
| FLJ_5 | 6.38 | 0.99 | 127.78 |
| FLJ_6 | 6.58 | 0.99 | 185.91 |
| CON_1 | 6.32 | 0.98 | 96.80 |
| CON_2 | 6.44 | 0.99 | 109.42 |
| CON_3 | 6.11 | 0.98 | 82.08 |
| CON_4 | 6.09 | 0.98 | 82.73 |
| CON_5 | 6.27 | 0.99 | 101.52 |
| CON_6 | 6.49 | 0.98 | 85.41 |

**Supplementary Table S6 PERMANOVA analysis of cecal content microbial community composition and test of homogeneity of group dispersions**

| **Test type** | **degrees of freedom** | **variance** | **F.Model** | **R^2^** | **P** |
| --- | --- | --- | --- | --- | --- |
| PERMANOVA | 1, 10 | 0.119731 | 2.349710 | 0.190264 | 0.028 |
|  |  | 0.509559 |  | 0.809735 |  |
|  |  | 0.629290 |  | 1 |  |
| Within-group dispersion test | 1, 10 | 0.0007 | 0.161 | / | 0.698 |
|  |  | 0.045 |  |  |  |

**Supplementary Table S7 Top 20 significantly up- and down-regulated differential metabolites in positive ion mode**

| **Differential metabolites** | **Superclass** | **Log2 foldchange** |
| --- | --- | --- |
| 1-(2-nitrobenzylidenamino)-2,4-imidazolidinedione | Organoheterocyclic compounds | 4.029170673 |
| N-Acetylmannosamine | Organic oxygen compounds | 3.778114216 |
| Lys-Trp-Lys | Organic acids and derivatives | 3.466239468 |
| Ursolic acid methyl ester | Lipids and lipid-like molecules | 2.237766458 |
| Cis-4,10,13,16-docosatetraenoic acid | Lipids and lipid-like molecules | 2.235463078 |
| 12s-hydroxy-5z,8e,10e-heptadecatrienoic acid | Lipids and lipid-like molecules | 2.16651049 |
| .alpha.-L-Asp-L-Lys | Organic acids and derivatives | 2.109831688 |
| 1-(4-piperidinyl)-1,3-dihydro-2h-indol-2-one | Organoheterocyclic compounds | 2.065372632 |
| L-thyronine | Organic acids and derivatives | 2.019153275 |
| Cys-Pro-Arg | Organic acids and derivatives | 1.889076344 |
| Empenthrin | Lipids and lipid-like molecules | 1.885535776 |
| Desogestrel | Lipids and lipid-like molecules | 1.870565066 |
| Ritonavir | Organic acids and derivatives | 1.836249759 |
| Asn-Lys | Organic acids and derivatives | 1.827865695 |
| Cis-4,7,10,13,16,19-docosahexaenoic acid | Lipids and lipid-like molecules | 1.801104141 |
| Met-Arg | Undefined | 1.77814417 |
| 4-pyridoxic acid | Organoheterocyclic compounds | 1.747482067 |
| 6-ketoprostaglandin e1 | Lipids and lipid-like molecules | 1.728935818 |
| O-tolidine | Benzenoids | 1.719707635 |
| Pro-Tyr | Undefined | 1.691880634 |
| Coniferin | Organic oxygen compounds | -4.609102531 |
| 1h-benzimidazole-7-carboxamide, 2-[(2r)-2-methyl-2-pyrrolidinyl]- | Undefined | -3.942114317 |
| 7-methoxy-2-methylquinolin-4-ol | Organoheterocyclic compounds | -3.670006709 |
| Pro-Ala | Undefined | -2.96768837 |
| Indole-2-carboxylic acid | Undefined | -2.450938291 |
| .alpha.-d-mannose pentaacetate | Organic acids and derivatives | -3.305692922 |
| Arthrobactin | Lipids and lipid-like molecules | -3.034836862 |
| Ala-Ala | Organoheterocyclic compounds | -3.03092535 |
| Pro-Ala | Undefined | -2.96768837 |
| D-Mannose | Organic oxygen compounds | -2.728956454 |
| 10-deacetylbaccatin iii | Lipids and lipid-like molecules | -2.468120344 |
| Indole-2-carboxylic acid | Undefined | -2.450938291 |
| Nordihydrocapsaicin | Benzenoids | -2.421946216 |
| 6-ketoprostaglandin f1.alpha. | Lipids and lipid-like molecules | -2.303441581 |
| 1h-indole-3-carboxamide, 1-(5-fluoropentyl)-n-1-naphthalenyl- | Organoheterocyclic compounds | -2.194849522 |
| Withaferin a | Lipids and lipid-like molecules | -2.16949705 |
| Tyr-Met | Undefined | -2.166639273 |
| 4-(1-piperazinyl)-1h-indole | Organoheterocyclic compounds | -2.125308307 |
| (4r)-4-((3r,5r,6s,7r,9s,10r,12s,13r,17r)-3,6,7,12-tetrahydroxy-10,13-dimethylhexadecahydro-1h-cyclopenta[a]phenanthren-17-yl)pentanoic acid | Lipids and lipid-like molecules | -2.117388941 |
| Curcumin | Phenylpropanoids and polyketides | -2.092563962 |

**Supplementary Table S8 Top 20 significantly up- and downregulated differential metabolites in negative ion mode**

| **Differential metabolites** | **Superclass** | **Log2 foldchange** |
| --- | --- | --- |
| P-toluenesulfonic acid | Benzenoids | 0.804261778 |
| 3-aminosalicylic acid | Benzenoids | 1.691283037 |
| (z)-5,8,11-trihydroxyoctadec-9-enoic acid | Lipids and lipid-like molecules | 0.269593173 |
| Erucic acid | Lipids and lipid-like molecules | 0.488419519 |
| Arachidic acid | Lipids and lipid-like molecules | 0.813235391 |
| 9,10-dihydroxy-12z-octadecenoic acid | Lipids and lipid-like molecules | 0.90701965 |
| Prostaglandin k1 | Lipids and lipid-like molecules | 1.01975396 |
| Azelaic acid | Lipids and lipid-like molecules | 1.077865736 |
| Octadecanedioic acid | Lipids and lipid-like molecules | 1.080272762 |
| Iloprost | Lipids and lipid-like molecules | 1.279115357 |
| 21-Hydroxypregnenolone | Lipids and lipid-like molecules | 1.345241439 |
| Prostaglandin e3 | Lipids and lipid-like molecules | 1.403118046 |
| Behenic acid | Lipids and lipid-like molecules | 1.404811799 |
| 20-hydroxy-4z,7z,10z,13z,16z,18e-docosahexaenoic acid | Lipids and lipid-like molecules | 1.497755036 |
| Progesterone | Lipids and lipid-like molecules | 1.604572228 |
| 15-cyclohexylpentanorprostaglandin f2.alpha. | Lipids and lipid-like molecules | 1.753567198 |
| 11beta-hydroxyprogesterone | Lipids and lipid-like molecules | 1.803741563 |
| Uridine | Nucleosides, nucleotides, and analogues | 0.717642747 |
| His-ser | Nucleosides, nucleotides, and analogues | 0.930279657 |
| Glutamine | Organic acids and derivatives | 1.811379202 |
| Biphenylindanone a | Benzenoids | -3.514431874 |
| Androsterone glucuronide | Lipids and lipid-like molecules | -3.547884783 |
| Estrone glucuronide | Lipids and lipid-like molecules | -2.345627873 |
| Taurochenodeoxycholate | Lipids and lipid-like molecules | -2.275952666 |
| 3.alpha.,7.alpha.-dihydroxy-12-oxocholanoic acid | Lipids and lipid-like molecules | -2.264068589 |
| Taurolithocholic acid sulfate | Lipids and lipid-like molecules | -2.248884555 |
| 17-trifluoromethylphenyltrinorprostaglandin f2.alpha. | Lipids and lipid-like molecules | -2.237702429 |
| Benzoic acid, 4-[[(1r,2e,4e,6z,9z)-1-[(1s)-4-carboxy-1-hydroxybutyl]-2,4,6,9-pentadecatetraen-1-yl]thio]- | Lipids and lipid-like molecules | -2.150477014 |
| 2-oleoyl-1-palmitoyl-sn-glycero-3-phosphoserine | Lipids and lipid-like molecules | -2.08224762 |
| Chenodeoxycholate | Lipids and lipid-like molecules | -2.050764681 |
| Arenobufagin | Lipids and lipid-like molecules | -1.953637331 |
| 1,3,5(10)-estratrien-3,17.beta.-diol 17-glucosiduronate | Lipids and lipid-like molecules | -1.865059416 |
| Sulfobacin b | Lipids and lipid-like molecules | -1.736128865 |
| 9-deoxy-9-methylene-16,16-dimethylprostaglandin e2 | Lipids and lipid-like molecules | -1.610177992 |
| 3-methoxyprostaglandin f1.alpha. | Lipids and lipid-like molecules | -1.481557622 |
| Leukotriene f4 | Lipids and lipid-like molecules | -1.443662525 |
| Deoxycholic acid | Lipids and lipid-like molecules | -1.356443379 |
| N-palmitoyl-d-erythro-dihydroceramide-1-phosphate | Lipids and lipid-like molecules | -1.345813435 |
| Linoleic acid | Lipids and lipid-like molecules | -1.099994396 |
| 3-oxocholic acid | Lipids and lipid-like molecules | -1.083241288 |

**Figure**


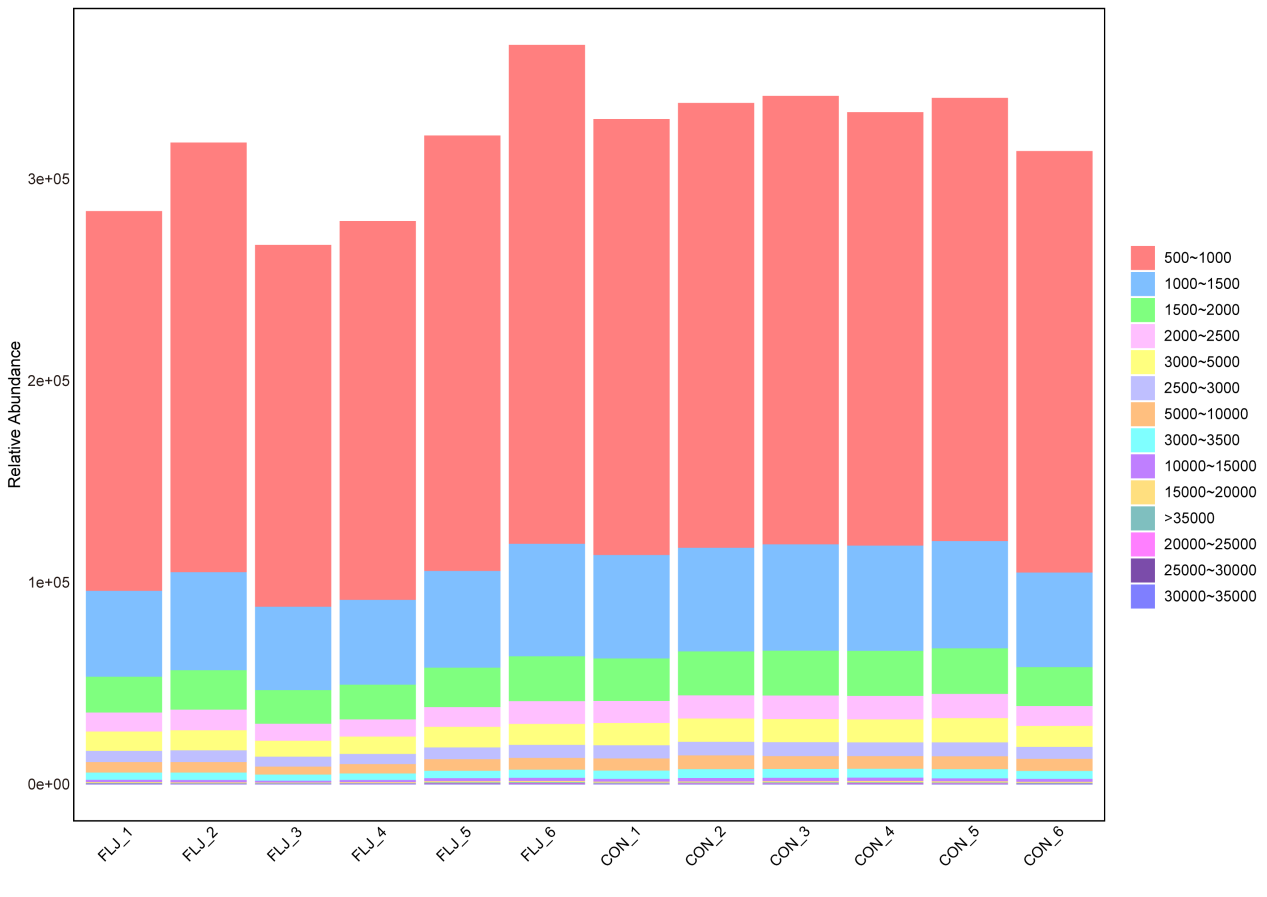


**Supplementary Figure S1** Contig length distribution.


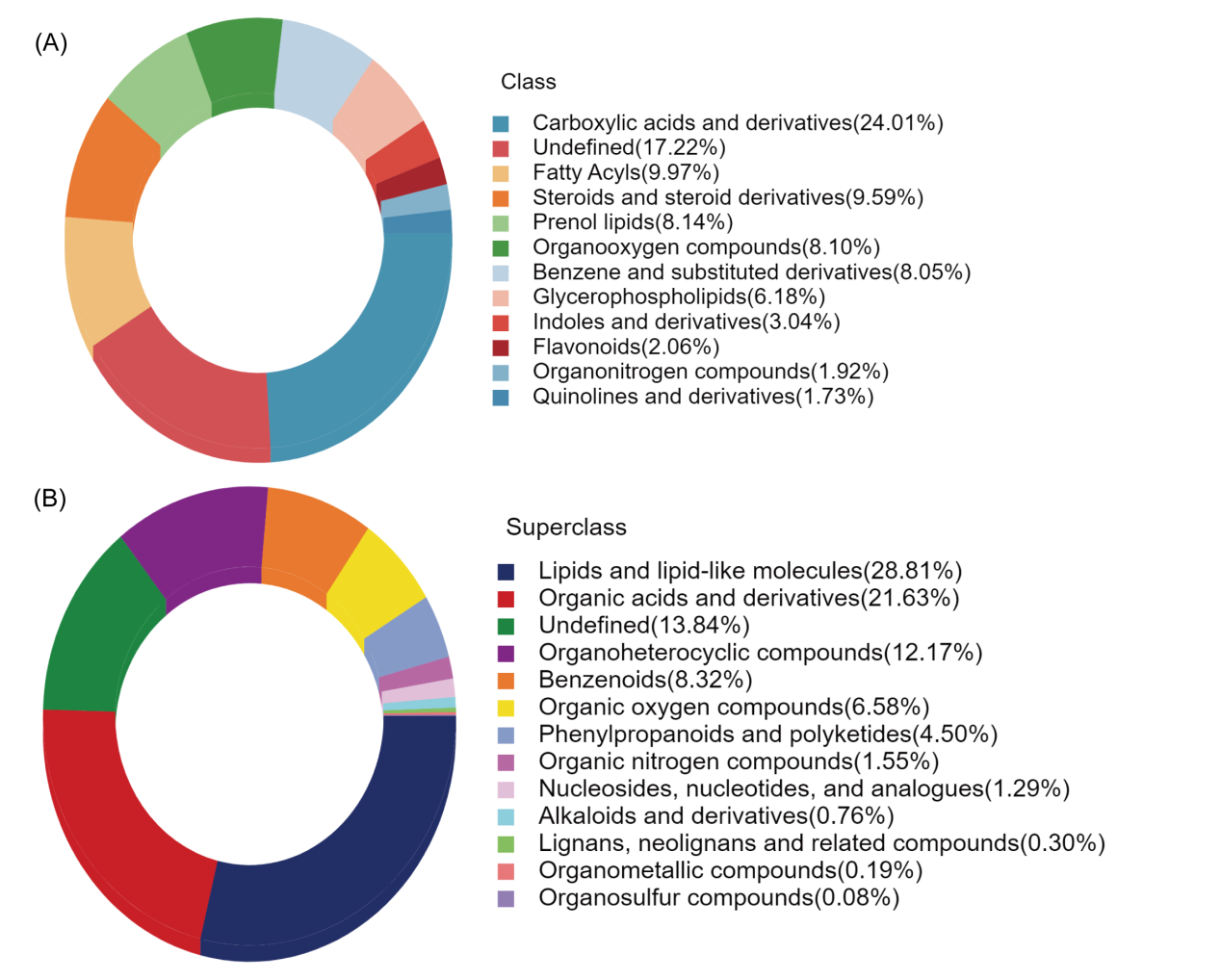


**Supplementary Figure S2 Metabolite composition.** (A). Class level types and proportions. (B). Superclass types and proportions
